# Supplementary material for: Genome-Wide Association and Genomic Prediction of Alfalfa (Medicago sativa L.) Biomass Yield Under Drought Stress
Source: Int J Mol Sci. 2025 Jan 13;26(2):608. doi: 10.3390/ijms26020608 (PMC11765341; doi:10.3390/ijms26020608)
Supplement: Supplementary file 1 [file ijms-26-00608-s001.zip › ijms-3398041-supplementary.pdf]

Supplementary Figures

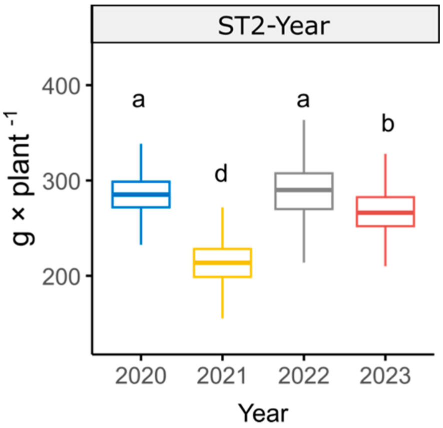

Figure S1. Predicted values of biomass yield modeled by year. Different letters indicate significantly different means (p-value < 0.05) based on Tukey's pairwise comparisons.

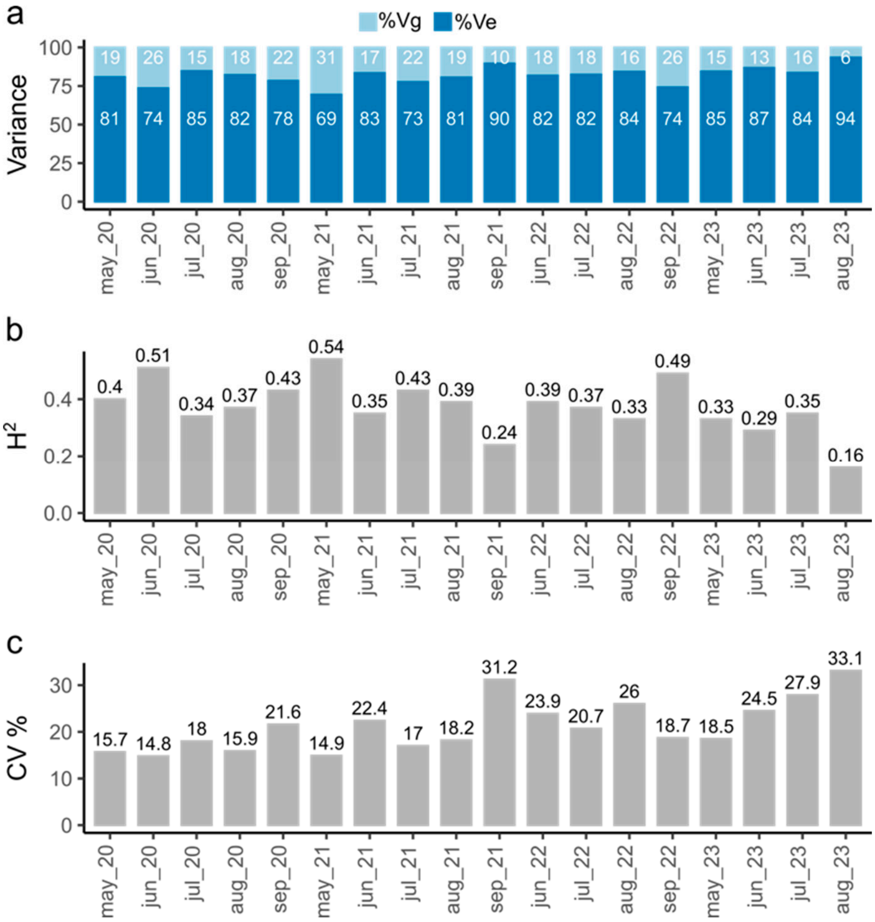

Figure S2. Variance components of biomass yield by single stage modeling. a. Percentage of variance components in 18 harvests. **b.** Broad sense heritability (H^2) calculated using the SpATS method [1]. **c.** Coefficient of variation (CV) in 18 harvests.

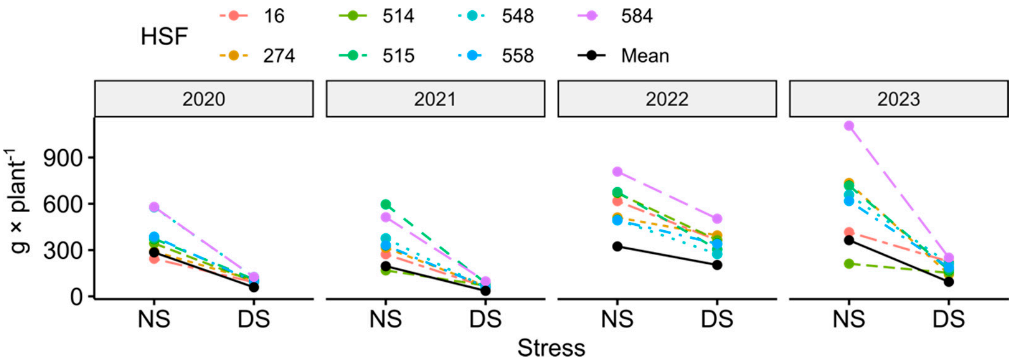

Figure S3. Biomass yield reduction in high-yielding half-sib families (HSFs). Seven HSFs with high yields under drought stress (DS) conditions were compared to the control grown under non-stress (NS) conditions over four years. NS harvests were May 2020, 2021, 2023, and June 2022, while DS harvests were September 2020, 2021, 2022, and August 2023. Black dot and line correspond to yield mean value.

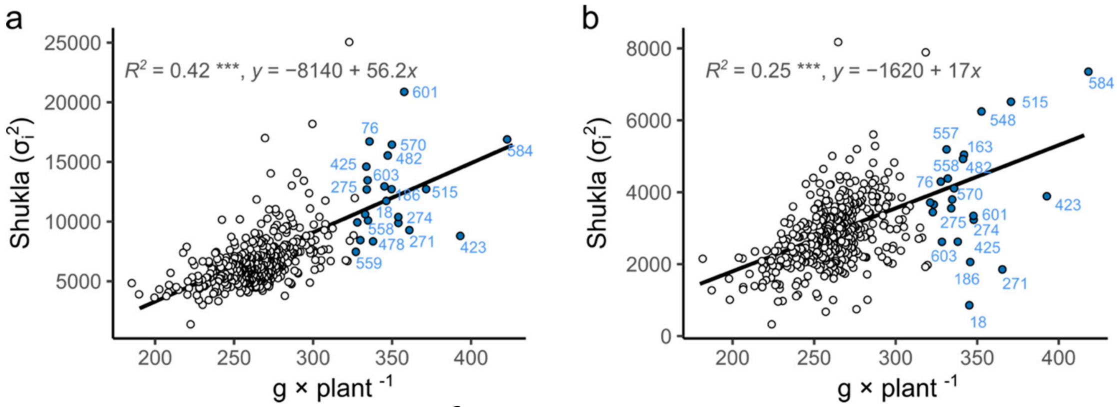

Figure S4. Shukla's stability variance (σ_i^2) in biomass yield. a. Stability variance using biomass yield by single harvest in 18 different alfalfa harvests. **b.** Stability variance using biomass yield averaged by month using five different months. Blue labels correspond to top 5% half-sib families by biomass yield. R^2 corresponds to the adjusted coefficient of determination of the regression, and asterisks (***) correspond to p-value < 0.001 of the fitted regression.

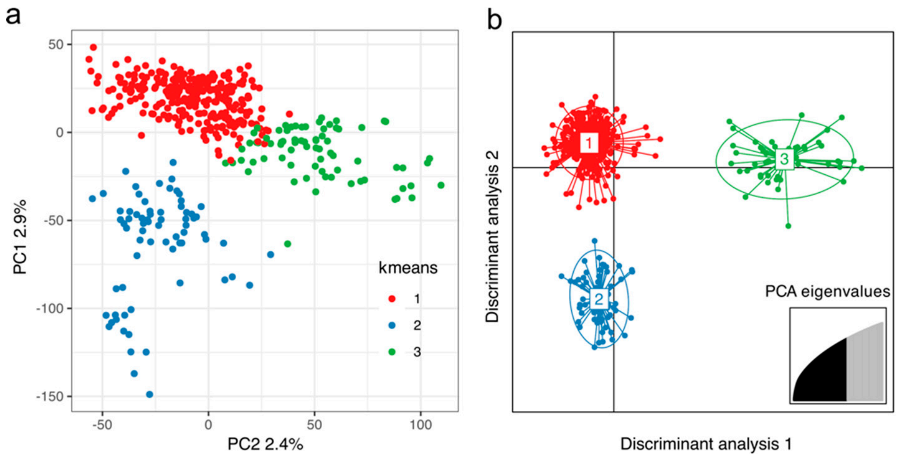

Figure S5. Population Structure of 424 alfalfa backcross half-sib families. a. Two-dimensional scatter plot plotting the first two principal components. Genotypes were clustered and colored by k-means = 3. **b.** Discriminant Analysis of Principal Component (DAPC) scatter plot. The axes represent the first two linear discriminants. Each oval represents a numbered cluster, and each dot represents a genotype. The inset chart indicates the cumulative variation retained (80%) during the analysis to describe the relationship between the clusters.

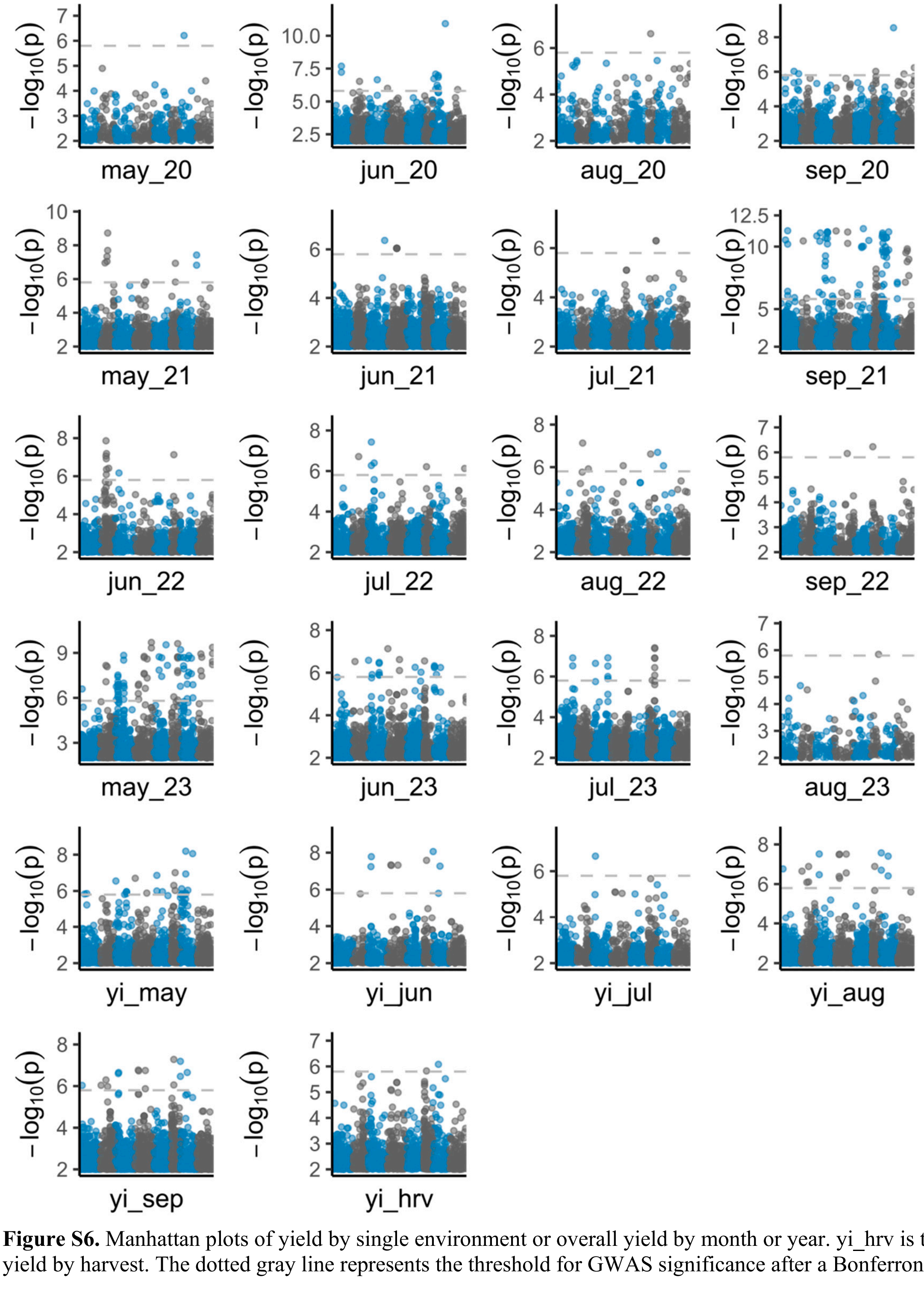

Figure S6. Manhattan plots of yield by single environment or overall yield by month or year. yi_hrv is the overall yield by harvest. The dotted gray line represents the threshold for GWAS significance after a Bonferroni correction.

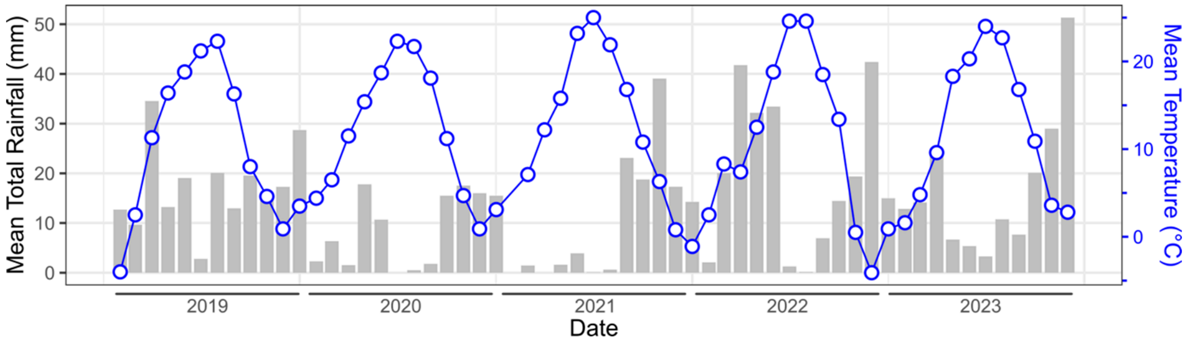

Figure S7. Weather conditions at the Roza experimental field in Prosser, WA. The bar plot represents the mean monthly total rainfall (in mm) in gray, with the scale on the left. The line and dot plot represent the mean monthly temperature (in ºC) in blue, with the scale on the right.

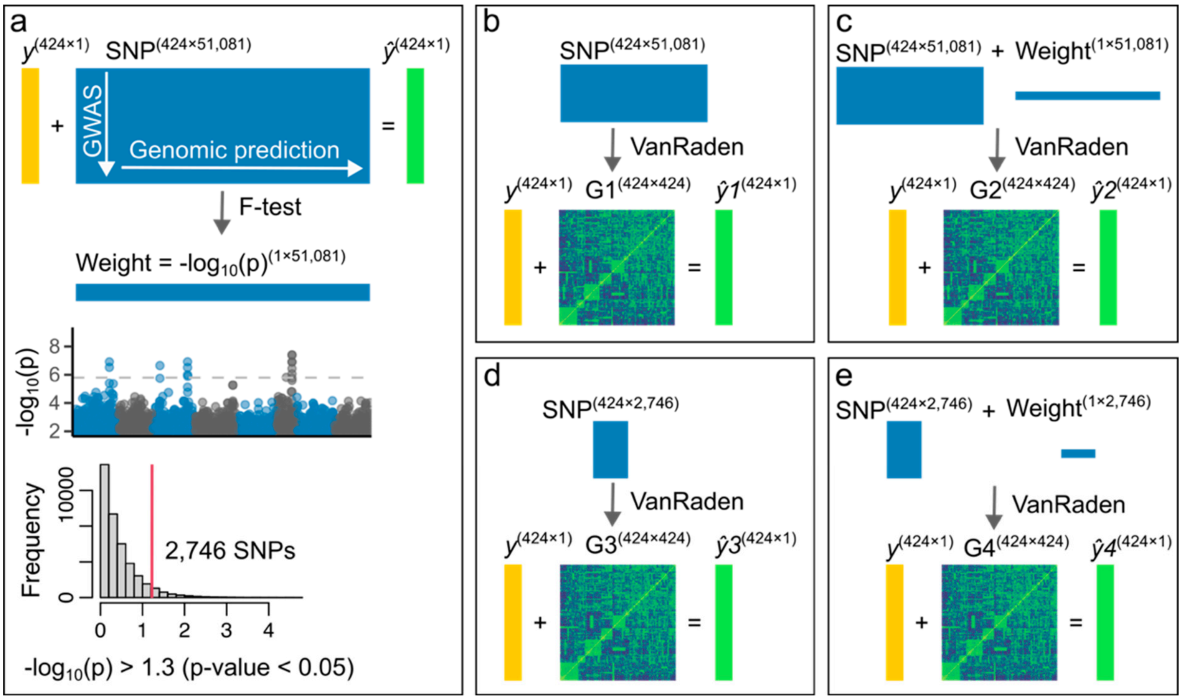

Figure S8. Generation of Genomic Relationship Matrices. a. Representation of GWAS and genomic prediction (GP) approaches. GP uses all genotypic information to obtain the genomic estimated breeding value (GEVB or ŷ), while GWAS computes the p-value for each marker using an F-test where the null hypothesis is that the SNP effect is zero. The vector of p-values is -log10 transformed and plotted as a Manhattan plot. Most of the markers accept the null hypothesis, retaining 2,746 SNPs with a p-value < 0.05. **b.** G1 matrix was generated using all markers. **c.** G2 matrix generated using all markers and weights from −log10(p-values) from the additive model between SNP markers and the overall predicted yield. **d.** G3 matrix was generated using only 2,746 SNPs with a −log10(p) > 1.3 (i.e., p-value < 0.05) from the GWAS additive model. **e.** G4 matrix was generated using the SNPs and weights from markers with a −log10(p) > 1.3.

Supplementary Tables

Table S1. Biomass yield information. Env, correspond to 18 different harvests from May to September for four years in a four or five cutting system. Date corresponds to the harvest date in a year-month-day format; water = 1 corresponds irrigation; miss and miss% correspond to number and percentage of missing plots.

ID	Env	Month	Year	Harv	Date	Water	miss	miss%
1	may_20	May	2020	1	2020-04-28	1	5	0.38
2	jun_20	June	2020	2	2020-06-02	1	7	0.54
3	jul_20	July	2020	3	2020-07-01	0	5	0.38
4	aug_20	August	2020	4	2020-08-05	0	5	0.38
5	sep_20	September	2020	5	2020-09-01	0	5	0.38
6	may_21	May	2021	1	2021-05-18	1	7	0.54
7	jun_21	June	2021	2	2021-06-16	1	7	0.54
8	jul_21	July	2021	3	2021-07-13	0	9	0.69
9	aug_21	August	2021	4	2021-08-10	0	10	0.76
10	sep_21	September	2021	5	2021-09-16	0	10	0.76
11	jun_22	June	2022	1	2022-05-23	1	14	1.07
12	jul_22	July	2022	2	2022-06-28	0	15	1.15
13	aug_22	August	2022	3	2022-08-01	0	18	1.38
14	sep_22	September	2022	4	2022-08-29	0	17	1.30
15	may_23	May	2023	1	2023-05-22	1	29	2.22
16	jun_23	June	2023	2	2023-06-20	1	26	1.99
17	jul_23	July	2023	3	2023-07-17	0	26	1.99
18	aug_23	August	2023	4	2023-08-24	0	28	2.14

Table S2. Population Information and Cluster Membership. VCF ID corresponds to the identifier in the VCF file; Sample ID corresponds to the identifier in the phenotypic data; Plant ID corresponds to the unique identifier for each half-sib family (HSF); BC_ID represents the parents used in the backcrossing, with the first denoting the female and the second the male parent. S and F1 represent the susceptible and F1 parent used in backcrossing, respectively. Half-sib families were clustered using k-means (K) and Discriminant Analysis of Principal Components (DAPC).

ID	VCF ID	Sample ID	Plant ID	BC_ID	S parent	F1 parent	K	DAPC
1	1_S502_R1_001	1_10B-75A-	1	1613-10×1622-10B	1613-10	1622-10B	1	1
2	4_S444_R1_001	4_10D-77A-14	4	1613-10×1622-10D	1613-10	1622-10D	2	2
3	5_S1_R1_001	5_10D-77A-11	5	1613-10×1622-10D	1613-10	1622-10D	3	1
4	6_S6_R1_001	6_10D-77A-12	6	1613-10×1622-10D	1613-10	1622-10D	3	3
5	7_S456_R1_001	7_10E-78A-1	7	1613-10×1622-10E	1613-10	1622-10E	2	2
6	9_S11_R1_001	9_11F-86A-2	9	1613-11×1622-11F	1613-11	1622-11F	1	1
7	12_S468_R1_001	12_11B-82A-10	12	1613-11×1622-11B	1613-11	1622-11B	2	2
8	13_S16_R1_001	13_11B-82A-18	13	1613-11×1622-11B	1613-11	1622-11B	1	1
9	14_S20_R1_001	14_11F-86A-11	14	1613-11×1622-11F	1613-11	1622-11F	1	1
10	15_S24_R1_001	15_13A-95A-1	15	1613-13×1622-13A	1613-13	1622-13A	1	1
11	16_S28_R1_001	16_13C-96A-1	16	1613-13×1622-13C	1613-13	1622-13C	1	1

12	17_S480_R1_001	17_13D-97A-1	17	1613-13×1622-13D	1613-13	1622-13D	1	1
13	18_S32_R1_001	18_13D-97A-2	18	1613-13×1622-13D	1613-13	1622-13D	1	1
14	19_S2_R1_001	19_13F-99A-2	19	1613-13×1622-13F	1613-13	1622-13F	1	1
15	20_S7_R1_001	20_14C-104A-8	20	1613-14×1622-14C	1613-14	1622-14C	1	1
16	22_S12_R1_001	22_14K-107A-2	22	1613-14×1622-14K	1613-14	1622-14K	1	1
17	23_S17_R1_001	23_15I-114A-1	23	1613-15×1622-15I	1613-15	1622-15I	2	2
18	24_S21_R1_001	24_15A-108A-1	24	1613-15×1622-15A	1613-15	1622-15A	2	2
19	25_S25_R1_001	25_15A-108A-9	25	1613-15×1622-15A	1613-15	1622-15A	2	2
20	26_S29_R1_001	26_15B-109A-1	26	1613-15×1622-15B	1613-15	1622-15B	2	2
21	27_S491_R1_001	27_15D-110A-1	27	1613-15×1622-15D	1613-15	1622-15D	2	2
23	29_S3_R1_001	29_15G-113A-15	29	1613-15×1622-15G	1613-15	1622-15G	2	2
24	30_S8_R1_001	30_15G-113A-1	30	1613-15×1622-15G	1613-15	1622-15G	2	2
27	34_S434_R1_001	34_15-113B-2	34	1613-15G×1622-15	1613-15	1622-15G	2	2
28	36_S458_R1_001	36_15G-113A-8	36	1613-15×1622-15G	1613-15	1622-15G	1	1
29	37_S423_R1_001	37_16E-121A-1	37	1613-16×1622-16E	1613-16	1622-16E	3	3
30	40_S459_R1_001	40_16D-120A-1	40	1613-16×1622-16D	1613-16	1622-16D	3	3
31	41_S470_R1_001	41_16D-120A-2	41	1613-16×1622-16D	1613-16	1622-16D	3	3
32	42_S482_R1_001	42_16E-121A-2	42	1613-16×1622-16E	1613-16	1622-16E	3	3
33	44_S505_R1_001	44_17E-129A-1	44	1613-17×1622-17E	1613-17	1622-17E	3	3
34	46_S483_R1_001	46_17F-130A-1	46	1613-17×1622-17F	1613-17	1622-17F	1	1
35	47_S494_R1_001	47_17F-130A-2	47	1613-17×1622-17F	1613-17	1622-17F	1	1
36	48_S13_R1_001	48_17L-136A-	48	1613-17×1622-17L	1613-17	1622-17L	1	1
37	49_S18_R1_001	49_18E-141A-1	49	1613-18×1622-18E	1613-18	1622-18E	3	3
38	50_S22_R1_001	50_18A-137A-8	50	1613-18×1622-18A	1613-18	1622-18A	3	3
39	51_S26_R1_001	51_18C-139A-2	51	1613-18×1622-18C	1613-18	1622-18C	3	3
40	52_S30_R1_001	52_18C-139A-8	52	1613-18×1622-18C	1613-18	1622-18C	3	3
41	53_S34_R1_001	53_18D-140A-1	53	1613-18×1622-18D	1613-18	1622-18D	3	3
42	54_S4_R1_001	54_18D-140A-2	54	1613-18×1622-18D	1613-18	1622-18D	3	3
43	55_S9_R1_001	55_18E-141A-2	55	1613-18×1622-18E	1613-18	1622-18E	3	3
44	56_S14_R1_001	56_18F-142A-1	56	1613-18×1622-18F	1613-18	1622-18F	3	3
45	57_S19_R1_001	57_18F-142A-2	57	1613-18×1622-18F	1613-18	1622-18F	1	1
46	59_S23_R1_001	59_19C-149A-1	59	1613-19×1622-19C	1613-19	1622-19C	1	1
47	60_S27_R1_001	60_19C-149A-2	60	1613-19×1622-19C	1613-19	1622-19C	1	1
48	62_S31_R1_001	62_2-4A-1	62	1613-2×1622-2D	1613-2	1622-2D	1	1
49	63_S36_R1_001	63_2C-3A-2	63	1613-2×1622-2C	1613-2	1622-2C	1	1
50	64_S47_R1_001	64_2D-4A-2	64	1613-2×1622-2D	1613-2	1622-2D	1	1
51	65_S59_R1_001	65_2E-5A-2	65	1613-2×1622-2E	1613-2	1622-2E	1	1
52	66_S71_R1_001	66_2L-9A-1	66	1613-2×1622-2L	1613-2	1622-2L	1	1
53	67_S83_R1_001	67_20H-161A-2	67	1613-20×1622-20H	1613-20	1622-20H	1	1
54	68_S95_R1_001	68_20H-161A-17-1	68	1613-20×1622-20H	1613-20	1622-20H	1	1

55	69_S106_R1_001	69_20H-161A-2	69	1613-20×1622-20H	1613-20	1622-20H	1	1
56	70_S118_R1_001	70_21A-166A-2	70	1613-21×1622-21A	1613-21	1622-21A	1	1
57	72_S37_R1_001	72_21G-169A-1	72	1613-21×1622-21G	1613-21	1622-21G	2	2
58	74_S48_R1_001	74_21K-171A-9	74	1613-21×1622-21K	1613-21	1622-21K	2	2
59	76_S60_R1_001	76_21K-171A-1	76	1613-21×1622-21K	1613-21	1622-21K	3	3
60	77_S72_R1_001	77_22I-179A-1	77	1613-22×1622-22I	1613-22	1622-22I	1	1
61	79_S84_R1_001	79_22I-179A-2	79	1613-22×1622-22I	1613-22	1622-22I	1	1
62	81_S96_R1_001	81_22F-176A-2	81	1613-22×1622-22F	1613-22	1622-22F	1	1
63	82_S107_R1_001	82_22F-176A-8	82	1613-22×1622-22F	1613-22	1622-22F	2	2
64	83_S119_R1_001	83_22F-176A-9	83	1613-22×1622-22F	1613-22	1622-22F	1	1
65	85_S38_R1_001	85_22H-178A-2	85	1613-22×1622-22H	1613-22	1622-22H	1	1
66	87_S49_R1_001	87_23C-183A-10	87	1613-23×1622-23C	1613-23	1622-23C	1	1
67	88_S61_R1_001	88_23C-183A-12	88	1613-23×1622-23C	1613-23	1622-23C	1	1
68	89_S73_R1_001	89_23D-184A-1	89	1613-23×1622-23D	1613-23	1622-23D	1	1
69	90_S85_R1_001	90_23D-184A-2	90	1613-23×1622-23D	1613-23	1622-23D	1	1
70	92_S97_R1_001	92_25I-189A-	92	1613-25×1622-25I	1613-25	1622-25I	1	1
71	93_S108_R1_001	93_25I-189A-1	93	1613-25×1622-25I	1613-25	1622-25I	3	3
72	94_S120_R1_001	94_26A-192A-1	94	1613-26×1622-26A	1613-26	1622-26A	1	1
73	95_S39_R1_001	95_26A-192A-2	95	1613-26×1622-26A	1613-26	1622-26A	1	1
74	96_S50_R1_001	96_26D-195A-9	96	1613-26×1622-26D	1613-26	1622-26D	1	1
75	97_S62_R1_001	97_26D-195A-13	97	1613-26×1622-26D	1613-26	1622-26D	1	1
76	98_S74_R1_001	98_26D-195A-15	98	1613-26×1622-26D	1613-26	1622-26D	1	1
77	100_S86_R1_001	100_26D-195A-16	100	1613-26×1622-26D	1613-26	1622-26D	1	1
79	104_S109_R1_001	104_28B-204A-1	104	1613-28×1622-28B	1613-28	1622-28B	3	3
80	107_S121_R1_001	107_28D-206A-9	107	1613-28×1622-28D	1613-28	1622-28D	3	3
81	108_S40_R1_001	108_28D-206A-11	108	1613-28×1622-28D	1613-28	1622-28D	3	3
82	111_S51_R1_001	111_29C-213A-2	111	1613-29×1622-29C	1613-29	1622-29C	1	1
83	112_S63_R1_001	112_2-4B-1	112	1622-2D×1613-2	1613-2	1622-2D	1	1
84	113_S75_R1_001	113_3C-11A-2	113	1613-3×1622-3C	1613-3	1622-3C	1	1
85	115_S87_R1_001	115_3D-12A-2	115	1613-3×1622-3D	1613-3	1622-3D	1	1
86	116_S98_R1_001	116_3F-13A-	116	1613-3×1622-3F	1613-3	1622-3F	2	2
87	117_S110_R1_001	117_3H-14A-2	117	1613-3×1622-3H	1613-3	1622-3H	1	1
88	118_S122_R1_001	118_3J-16A-1	118	1613-3×1622-3J	1613-3	1622-3J	1	1
91	121_S64_R1_001	121_30A-218A-8	121	1613-30×1622-30A	1613-30	1622-30A	1	1
92	123_S76_R1_001	123_30E-222A-2	123	1613-30×1622-30E	1613-30	1622-30E	3	3
93	124_S88_R1_001	124_30L-228A-2	124	1613-30×1622-30L	1613-30	1622-30L	1	1
94	125_S99_R1_001	125_30B-219A-2	125	1613-30×1622-30B	1613-30	1622-30B	1	1
95	126_S111_R1_001	126_32D-236A-	126	1613-32×1622-32D	1613-32	1622-32D	2	2
96	128_S123_R1_001	128_4B-18A-1	128	1613-4×1622-4B	1613-4	1622-4B	1	1
97	129_S41_R1_001	129_4C-19A-2	129	1613-4×1622-4C	1613-4	1622-4C	1	1

98	130_S53_R1_001	130_4D-20A-8	130	1613-4×1622-4D	1613-4	1622-4D	1	1
99	131_S65_R1_001	131_4G-23A-1	131	1613-4×1622-4G	1613-4	1622-4G	1	1
100	133_S77_R1_001	133_4L-27A-2	133	1613-4×1622-4L	1613-4	1622-4L	1	1
101	134_S89_R1_001	134_4L-27A-	134	1613-4×1622-4L	1613-4	1622-4L	1	1
102	135_S100_R1_001	135_5A-28A-1	135	1613-5×1622-5A	1613-5	1622-5A	1	1
103	138_S112_R1_001	138_5I-34A-8	138	1613-5×1622-5I	1613-5	1622-5I	1	1
104	140_S124_R1_001	140_5B-29A-2	140	1613-5×1622-5B	1613-5	1622-5B	1	1
105	141_S42_R1_001	141_5E-31A-1	141	1613-5×1622-5E	1613-5	1622-5E	1	1
106	143_S54_R1_001	143_5F-32A-1	143	1613-5×1622-5F	1613-5	1622-5F	1	1
107	144_S66_R1_001	144_5F-32A-2	144	1613-5×1622-5F	1613-5	1622-5F	2	2
108	145_S78_R1_001	145_5G-33A-2	145	1613-5×1622-5G	1613-5	1622-5G	3	3
109	146_S90_R1_001	146_5L-37A-	146	1613-5×1622-5L	1613-5	1622-5L	1	1
110	147_S101_R1_001	147_6A-38A-1	147	1613-6×1622-6A	1613-6	1622-6A	3	3
111	149_S113_R1_001	149_6F-42A-8-1	149	1613-6×1622-6F	1613-6	1622-6F	3	3
112	150_S125_R1_001	150_6F-42A-8-2	150	1613-6×1622-6F	1613-6	1622-6F	1	1
113	151_S43_R1_001	151_6H-44A-2	151	1613-6×1622-6H	1613-6	1622-6H	3	3
114	152_S55_R1_001	152_6L-47A-2	152	1613-6×1622-6L	1613-6	1622-6L	1	1
115	153_S67_R1_001	153_6L-47A-9	153	1613-6×1622-6L	1613-6	1622-6L	3	3
116	155_S79_R1_001	155_17A-126A-2	155	1613-7×1622-17A	1613-7	1622-17A	1	1
117	156_S91_R1_001	156_7A-48A-1	156	1613-7×1622-7A	1613-7	1622-7A	2	2
118	157_S102_R1_001	157_7A-48A-2	157	1613-7×1622-7A	1613-7	1622-7A	1	1
119	158_S114_R1_001	158_7B-49A-8	158	1613-7×1622-7B	1613-7	1622-7B	2	2
120	160_S126_R1_001	160_8B-59A-1	160	1613-8×1622-8B	1613-8	1622-8B	1	1
121	161_S44_R1_001	161_8-64A-2	161	1613-8×1622-8K	1613-8	1622-8K	1	1
122	163_S56_R1_001	163_8A-58A-1	163	1613-8×1622-8A	1613-8	1622-8A	1	1
123	164_S68_R1_001	164_9F-69A-9	164	1613-9×1622-9F	1613-9	1622-9F	1	1
124	166_S80_R1_001	166_1-2B-1	166	1622-1K×1613-1	1613-1	1622-1K	1	1
125	168_S92_R1_001	168_10-76B-9	168	1622-10C×1613-10	1613-10	1622-10C	2	2
126	169_S10_R1_001	169_10-77B-8	169	1622-10D×1613-10	1613-10	1622-10D	1	1
127	170_S115_R1_001	170_10-77B-13	170	1622-10D×1613-10	1613-10	1622-10D	3	3
128	172_S127_R1_001	172_10-77B-2	172	1622-10D×1613-10	1613-10	1622-10D	1	1
129	173_S45_R1_001	173_10-81B-2	173	1622-10K×1613-10	1613-10	1622-10K	1	1
130	175_S57_R1_001	175_11-84B-1	175	1622-11D×1613-11	1613-11	1622-11D	1	1
131	176_S69_R1_001	176_11-82B-8	176	1622-11B×1613-11	1613-11	1622-11B	1	1
132	178_S81_R1_001	178_11-82B-13	178	1622-11B×1613-11	1613-11	1622-11B	3	3
133	183_S93_R1_001	183_11-87B-2	183	1622-11G×1613-11	1613-11	1622-11G	1	1
134	184_S104_R1_001	184_11-87B-	184	1622-11G×1613-11	1613-11	1622-11G	1	1
135	186_S116_R1_001	186_312-93B-1	186	1622-12E×1613-12	1613-12	1622-12E	1	1
136	187_S15_R1_001	187_313-101B-1	187	1622-13H×1613-13	1613-13	1622-13H	1	1
137	188_S5_R1_001	188_313-102B-1	188	1622-13I×1613-13	1613-13	1622-13I	1	1

138	189_S58_R1_001	189_313-102B-2	189	1622-13I×1613-13	1613-13	1622-13I	1	1
139	190_S70_R1_001	190_13-96B-2	190	1622-13C×1613-13	1613-13	1622-13C	1	1
140	191_S82_R1_001	191_13-100B-1	191	1622-13G×1613-13	1613-13	1622-13G	1	1
141	193_S94_R1_001	193_14-107B-1	193	1622-14K×1613-14	1613-14	1622-14K	2	2
142	194_S105_R1_001	194_14-103B-2	194	1622-14A×1613-14	1613-14	1622-14A	2	2
143	195_S117_R1_001	195_14-103B-8	195	1622-14A×1613-14	1613-14	1622-14A	1	1
144	196_S129_R1_001	196_14-104B-1	196	1622-14C×1613-14	1613-14	1622-14C	3	3
145	198_S130_R1_001	198_14-107B-2	198	1622-14K×1613-14	1613-14	1622-14K	3	3
146	199_S141_R1_001	199_15-108B-2	199	1622-15A×1613-15	1613-15	1622-15A	2	2
147	200_S153_R1_001	200_15-109B-1	200	1613-15×1622-15B	1613-15	1622-15B	2	2
148	201_S165_R1_001	201_15-109A-2	201	1622-15B×1613-15	1613-15	1622-15B	2	2
149	204_S176_R1_001	204_15-112B-11	204	1622-15F×1613-15	1613-15	1622-15F	2	2
150	206_S188_R1_001	206_16-118B-1	206	1622-16B×1613-16	1613-16	1622-16B	1	1
151	207_S200_R1_001	207_16-124B-1	207	1622-16I×1613-16	1613-16	1622-16I	3	3
152	208_S212_R1_001	208_16-124B-2	208	1622-16I×1613-16	1613-16	1622-16I	3	3
153	210_S131_R1_001	210_16-121B-1	210	1622-16E×1613-16	1613-16	1622-16E	1	1
154	211_S142_R1_001	211_16-121B-2	211	1622-16E×1613-16	1613-16	1622-16E	3	3
155	212_S154_R1_001	212_16-123B-8	212	1622-16H×1613-16	1613-16	1622-16H	3	3
156	213_S35_R1_001	213_17-133B-2	213	1622-17I×1613-17	1613-17	1622-17I	1	1
157	214_S177_R1_001	214_17-136B-1	214	1622-17L×1613-17	1613-17	1622-17L	1	1
158	215_S189_R1_001	215_17-133B-1	215	1622-17I×1613-17	1613-17	1622-17I	1	1
159	217_S201_R1_001	217_17-126B-9	217	1622-17A×1613-17	1613-17	1622-17A	1	1
161	219_S132_R1_001	219_17-132B-1	219	1622-17H×1613-17	1613-17	1622-17H	1	1
162	220_S143_R1_001	220_17-132B-2	220	1622-17H×1613-17	1613-17	1622-17H	1	1
163	223_S155_R1_001	223_18-137B-2	223	1622-18A×1613-18	1613-18	1622-18A	3	3
164	224_S166_R1_001	224_18-139B-1	224	1622-18C×1613-18	1613-18	1622-18C	3	3
165	225_S178_R1_001	225_18-139B-2	225	1622-18C×1613-18	1613-18	1622-18C	3	3
166	226_S190_R1_001	226_18-140B-1	226	1622-18D×1613-18	1613-18	1622-18D	3	3
167	227_S202_R1_001	227_18-141B-	227	1622-18E×1613-18	1613-18	1622-18E	3	3
168	229_S214_R1_001	229_19-152B-2	229	1622-19I×1613-19	1613-19	1622-19I	1	1
169	230_S503_R1_001	230_19-148B-2	230	1622-19B×1613-19	1613-19	1622-19B	1	1
170	231_S144_R1_001	231_19-148B-10	231	1622-19B×1613-19	1613-19	1622-19B	1	1
171	232_S156_R1_001	232_19-148B-12	232	1622-19B×1613-19	1613-19	1622-19B	1	1
172	233_S167_R1_001	233_19-154B-2	233	1622-19K×1613-19	1613-19	1622-19K	1	1
173	234_S179_R1_001	234_1-2B-2	234	1622-1K×1613-1	1613-1	1622-1K	3	3
174	235_S191_R1_001	235_2-8B-1	235	1622-2K×1613-2	1613-2	1622-2K	3	3
175	236_S203_R1_001	236_20-162B-2	236	1622-20I×1613-20	1613-20	1622-20I	1	1
176	238_S215_R1_001	238_20-161B-8	238	1622-20H×1613-20	1613-20	1622-20H	1	1
177	239_S133_R1_001	239_20-161B-9	239	1622-20H×1613-20	1613-20	1622-20H	1	1
178	240_S145_R1_001	240_20-161B-10	240	1622-20H×1613-20	1613-20	1622-20H	1	1

179	241_S157_R1_001	241_20-161B-12	241	1622-20H×1613-20	1613-20	1622-20H	1	1
180	242_S168_R1_001	242_20-164B-1	242	1622-20K×1613-20	1613-20	1622-20K	1	1
181	243_S180_R1_001	243_21-168B-1	243	1622-21C×1613-21	1613-21	1622-21C	3	1
182	244_S192_R1_001	244_21-168B-2	244	1622-21C×1613-21	1613-21	1622-21C	1	1
183	245_S204_R1_001	245_21-169B-1	245	1622-21G×1613-21	1613-21	1622-21G	1	1
184	247_S216_R1_001	247_22-176B-1	247	1622-22F×1613-22	1613-22	1622-22F	1	1
185	248_S134_R1_001	248_22-179B-1	248	1622-22I×1613-22	1613-22	1622-22I	1	1
186	249_S146_R1_001	249_22-173B-2	249	1622-22B×1613-22	1613-22	1622-22B	2	2
187	250_S158_R1_001	250_22-173B-	250	1622-22B×1613-22	1613-22	1622-22B	1	1
188	251_S169_R1_001	251_22-175B-2	251	1622-22E×1613-22	1613-22	1622-22E	1	1
189	252_S181_R1_001	252_22-176B-2	252	1622-22F×1613-22	1613-22	1622-22F	2	2
190	253_S193_R1_001	253_22-178B-1	253	1622-22H×1613-22	1613-22	1622-22H	1	1
191	255_S205_R1_001	255_23-184B-1	255	1622-23D×1613-23	1613-23	1622-23D	1	1
192	257_S217_R1_001	257_23-182B-10	257	1622-23A×1613-23	1613-23	1622-23A	2	2
193	258_S103_R1_001	258_23-183B-9	258	1622-23C×1613-23	1613-23	1622-23C	1	1
195	263_S159_R1_001	263_25-188B-1	263	1622-25C×1613-25	1613-25	1622-25C	1	1
196	264_S170_R1_001	264_26-194B-1	264	1622-26C×1613-26	1613-26	1622-26C	2	2
197	265_S182_R1_001	265_26-195B-1	265	1622-26D×1613-26	1613-26	1622-26D	1	1
198	266_S194_R1_001	266_26-198B-1	266	1622-26G×1613-26	1613-26	1622-26G	1	1
199	267_S206_R1_001	267_26-193B-1	267	1622-26B×1613-26	1613-26	1622-26B	1	1
200	268_S218_R1_001	268_26-194B-2	268	1622-26C×1613-26	1613-26	1622-26C	1	1
201	269_S136_R1_001	269_26-194B-9	269	1622-26C×1613-26	1613-26	1622-26C	1	1
202	271_S148_R1_001	271_26-195B-10	271	1622-26D×1613-26	1613-26	1622-26D	2	2
203	272_S160_R1_001	272_26-195B-15	272	1622-26D×1613-26	1613-26	1622-26D	1	1
204	273_S171_R1_001	273_26-195B-12	273	1622-26D×1613-26	1613-26	1622-26D	1	1
205	274_S183_R1_001	274_26-196B-2	274	1622-26E×1613-26	1613-26	1622-26E	1	1
206	275_S195_R1_001	275_26-196B-	275	1622-26E×1613-26	1613-26	1622-26E	3	3
207	280_S207_R1_001	280_29-217B-1	280	1622-29I×1613-29	1613-29	1622-29I	1	1
208	282_S219_R1_001	282_29-214B-2	282	1622-29D×1613-29	1613-29	1622-29D	1	1
209	284_S137_R1_001	284_2-3B-1	284	1622-2C×1613-2	1613-2	1622-2C	1	1
210	285_S149_R1_001	285_2-3B-2	285	1622-2C×1613-2	1613-2	1622-2C	1	1
211	286_S161_R1_001	286_2-4B-2-A	286	1622-2D×1613-2	1613-2	1622-2D	1	1
212	287_S172_R1_001	287_2-4B-2-B	287	1622-2D×1613-2	1613-2	1622-2D	1	1
213	288_S184_R1_001	288_2-5B-2	288	1622-2E×1613-2	1613-2	1622-2E	1	1
214	291_S196_R1_001	291_30-227B-1	291	1622-30K×1613-30	1613-30	1622-30K	1	1
215	295_S208_R1_001	295_30-222B-2	295	1622-30E×1613-30	1613-30	1622-30E	1	1
216	296_S220_R1_001	296_30-223B-	296	1622-30G×1613-30	1613-30	1622-30G	1	1
217	297_S138_R1_001	297_30-224B-2	297	1622-30H×1613-30	1613-30	1622-30H	1	1
218	298_S150_R1_001	298_30-227B-	298	1622-30K×1613-30	1613-30	1622-30K	1	1
219	299_S162_R1_001	299_30-228B-1	299	1622-30L×1613-30	1613-30	1622-30L	1	1

220	300_S173_R1_001	300_32-240B-1	300	1622-32H×1613-32	1613-32	1622-32H	1	1
221	302_S185_R1_001	302_32-244B-1	302	1622-32L×1613-32	1613-32	1622-32L	1	1
222	303_S197_R1_001	303_3-11B-2	303	1622-3C×1613-3	1613-3	1622-3C	1	1
223	304_S209_R1_001	304_3-11B-1	304	1622-3C×1613-3	1613-3	1622-3C	2	2
224	305_S221_R1_001	305_3-13B-2	305	1622-3F×1613-3	1613-3	1622-3F	1	1
225	306_S139_R1_001	306_3-13B-9	306	1622-3F×1613-3	1613-3	1622-3F	1	1
226	307_S151_R1_001	307_3-16B-2	307	1622-3J×1613-3	1613-3	1622-3J	1	1
227	308_S163_R1_001	308_3-16B-11	308	1622-3J×1613-3	1613-3	1622-3J	1	1
228	309_S174_R1_001	309_3-17B-2	309	1622-3K×1613-3	1613-3	1622-3K	1	1
229	311_S186_R1_001	311_4-19B-1	311	1622-4C×1613-4	1613-4	1622-4C	1	1
230	312_S198_R1_001	312_4-21B-8	312	1622-4E×1613-4	1613-4	1622-4E	1	1
231	314_S210_R1_001	314_4-24B-8	314	1622-4H×1613-4	1613-4	1622-4H	2	2
232	315_S222_R1_001	315_4-25B-2	315	1622-4J×1613-4	1613-4	1622-4J	1	1
233	316_S140_R1_001	316_4-25B-1	316	1613-4×1622-4J	1613-4	1622-4J	1	1
234	318_S152_R1_001	318_4-27B-1	318	1622-4L×1613-4	1613-4	1622-4L	1	1
235	319_S164_R1_001	319_5-34B-1	319	1622-5I×1613-5	1613-5	1622-5I	3	3
236	320_S175_R1_001	320_5-30B-1	320	1622-5D×1613-5	1613-5	1622-5D	2	2
237	321_S187_R1_001	321_5-31B-1	321	1622-5E×1613-5	1613-5	1622-5E	1	1
238	322_S199_R1_001	322_5-31B-2	322	1622-5E×1613-5	1613-5	1622-5E	1	1
239	324_S211_R1_001	324_5-33B-1	324	1622-5G×1613-5	1613-5	1622-5G	1	1
240	325_S223_R1_001	325_5-33B-2	325	1622-5G×1613-5	1613-5	1622-5G	1	1
241	326_S224_R1_001	326_6-47B-1	326	1622-6L×1613-6	1613-6	1622-6L	2	2
242	327_S236_R1_001	327_6-41B-1	327	1622-6D×1613-6	1613-6	1622-6D	1	1
244	330_S259_R1_001	330_6-43B-10	330	1622-6G×1613-6	1613-6	1622-6G	1	1
245	331_S128_R1_001	331_6-44B-1	331	1622-6H×1613-6	1613-6	1622-6H	1	1
247	333_S294_R1_001	333_6-47B-2	333	1622-6L×1613-6	1613-6	1622-6L	1	1
248	334_S306_R1_001	334_7-57B-1	334	1622-7L×1613-7	1613-7	1622-7L	1	1
249	335_S225_R1_001	335_7-48B-1	335	1622-7A×1613-7	1613-7	1622-7A	1	1
250	337_S237_R1_001	337_9-72B-1	337	1622-9I×1613-9	1613-9	1622-9I	1	1
251	338_S248_R1_001	338_9-67B-1	338	1622-9D×1613-9	1613-9	1622-9D	3	3
252	339_S260_R1_001	339_9-69B-1	339	1622-9F×1613-9	1613-9	1622-9F	1	1
253	342_S272_R1_001	342_9-70B-	342	1622-9G×1613-9	1613-9	1622-9G	1	1
254	343_S283_R1_001	343_10D-77A-7	343	1613-10×1622-10D	1613-10	1622-10D	1	1
255	344_S295_R1_001	344_11B-82A-4	344	1613-11×1622-11B	1613-11	1622-11B	1	1
256	350_S307_R1_001	350_14C-104A-4	350	1613-14×1622-14C	1613-14	1622-14C	1	1
257	351_S226_R1_001	351_14E-105A-4	351	1613-14×1622-14E	1613-14	1622-14E	1	1
258	353_S238_R1_001	353_14F-106A-3	353	1613-14×1622-14F	1613-14	1622-14F	1	1
259	354_S249_R1_001	354_15I-114A-4	354	1613-15×1622-15I	1613-15	1622-15I	2	2
260	355_S261_R1_001	355_15A-108A-6	355	1613-15×1622-15A	1613-15	1622-15A	2	2
261	356_S273_R1_001	356_15A-108A-3	356	1613-15×1622-15A	1613-15	1622-15A	2	2

262	357_S284_R1_001	357_15A-108A-7	357	1613-15×1622-15A	1613-15	1622-15A	2	2
263	358_S296_R1_001	358_15A-108A-4	358	1613-15×1622-15A	1613-15	1622-15A	2	2
264	360_S308_R1_001	360_15F-112A-3	360	1613-15×1622-15F	1613-15	1622-15F	2	2
265	361_S227_R1_001	361_15F-112A-6	361	1613-15×1622-15F	1613-15	1622-15F	2	2
266	362_S239_R1_001	362_15F-112A-7	362	1613-15×1622-15F	1613-15	1622-15F	2	2
267	363_S250_R1_001	363_15F-112A-5	363	1613-15×1622-15F	1613-15	1622-15F	2	2
268	364_S262_R1_001	364_15G-113A-4	364	1613-15×1622-15G	1613-15	1622-15G	2	2
269	366_S274_R1_001	366_17F-130A-5	366	1613-17×1622-17F	1613-17	1622-17F	2	2
270	367_S285_R1_001	367_17F-130A-6	367	1613-17×1622-17F	1613-17	1622-17F	1	1
271	368_S297_R1_001	368_17H-132A-6	368	1613-17×1622-17H	1613-17	1622-17H	1	1
272	369_S309_R1_001	369_17H-132A-4	369	1613-17×1622-17H	1613-17	1622-17H	3	3
273	370_S228_R1_001	370_18C-139A-4	370	1613-18×1622-18C	1613-18	1622-18C	3	3
274	371_S240_R1_001	371_18C-139A-6	371	1613-18×1622-18C	1613-18	1622-18C	3	3
275	372_S251_R1_001	372_18D-140A-6	372	1613-18×1622-18D	1613-18	1622-18D	3	3
276	373_S263_R1_001	373_18D-140A-7	373	1613-18×1622-18D	1613-18	1622-18D	3	3
277	374_S275_R1_001	374_18D-140A-4	374	1613-18×1622-18D	1613-18	1622-18D	3	3
278	375_S286_R1_001	375_18F-142A-3	375	1613-18×1622-18F	1613-18	1622-18F	3	3
279	376_S298_R1_001	376_18F-142A-4	376	1613-18×1622-18F	1613-18	1622-18F	3	3
280	378_S310_R1_001	378_19B-148A-3	378	1613-19×1622-19B	1613-19	1622-19B	1	1
281	379_S229_R1_001	379_19C-149A-7	379	1613-19×1622-19C	1613-19	1622-19C	1	1
282	380_S241_R1_001	380_19C-149A-4	380	1613-19×1622-19C	1613-19	1622-19C	1	1
283	381_S252_R1_001	381_19C-149A-5	381	1613-19×1622-19C	1613-19	1622-19C	1	1
284	382_S264_R1_001	382_19J-153A-6	382	1613-19×1622-19J	1613-19	1622-19J	1	1
285	383_S276_R1_001	383_19J-153A-5	383	1613-19×1622-19J	1613-19	1622-19J	1	1
286	384_S287_R1_001	384_19K-154A-3	384	1613-19×1622-19K	1613-19	1622-19K	1	1
287	385_S299_R1_001	385_19K-154A-5	385	1613-19×1622-19K	1613-19	1622-19K	1	1
288	386_S311_R1_001	386_2D-4A-4	386	1613-2×1622-2D	1613-2	1622-2D	2	2
289	387_S230_R1_001	387_2D-4A-3	387	1613-2×1622-2D	1613-2	1622-2D	1	1
290	389_S242_R1_001	389_20H-161A-3	389	1613-20×1622-20H	1613-20	1622-20H	1	1
291	390_S253_R1_001	390_20H-161A-6	390	1613-20×1622-20H	1613-20	1622-20H	1	1
292	391_S265_R1_001	391_20H-161A-7	391	1613-20×1622-20H	1613-20	1622-20H	1	1
293	392_S277_R1_001	392_21I-170A-4	392	1613-21×1622-21I	1613-21	1622-21I	1	1
294	393_S288_R1_001	393_21A-166A-3	393	1613-21×1622-21A	1613-21	1622-21A	3	3
295	397_S300_R1_001	397_22F-176A-4	397	1613-22×1622-22F	1613-22	1622-22F	1	1
296	400_S312_R1_001	400_22F-176A-7	400	1613-22×1622-22F	1613-22	1622-22F	1	1
297	401_S231_R1_001	401_22F-176A-3	401	1613-22×1622-22F	1613-22	1622-22F	1	1
298	402_S243_R1_001	402_22G-177A-3	402	1613-22×1622-22G	1613-22	1622-22G	1	1
299	404_S254_R1_001	404_23A-182A-4	404	1613-23×1622-23A	1613-23	1622-23A	1	1
300	405_S266_R1_001	405_23D-184A-6	405	1613-23×1622-23D	1613-23	1622-23D	3	1
301	406_S278_R1_001	406_23D-184A-7	406	1613-23×1622-23D	1613-23	1622-23D	3	3

302	408_S289_R1_001	408_23F-186A-7	408	1613-23×1622-23F	1613-23	1622-23F	1	1
303	409_S301_R1_001	409_26A-192A-4	409	1613-26×1622-26A	1613-26	1622-26A	2	2
304	410_S313_R1_001	410_26D-195A-3	410	1613-26×1622-26D	1613-26	1622-26D	1	1
305	411_S232_R1_001	411_26D-195A-4	411	1613-26×1622-26D	1613-26	1622-26D	1	1
306	413_S244_R1_001	413_26D-195A-8	413	1613-26×1622-26D	1613-26	1622-26D	1	1
307	414_S255_R1_001	414_26E-196A-5	414	1613-26×1622-26E	1613-26	1622-26E	1	1
308	416_S267_R1_001	416_26E-196A-4	416	1613-26×1622-26E	1613-26	1622-26E	3	3
309	420_S279_R1_001	420_28B-204A-5	420	1613-28×1622-28B	1613-28	1622-28B	1	1
310	421_S290_R1_001	421_28D-206A-3	421	1613-28×1622-28D	1613-28	1622-28D	2	2
311	422_S302_R1_001	422_28D-206A-6	422	1613-28×1622-28D	1613-28	1622-28D	1	1
312	423_S314_R1_001	423_28D-206A-7	423	1613-28×1622-28D	1613-28	1622-28D	1	1
313	425_S233_R1_001	425_28D-206A-4	425	1613-28×1622-28D	1613-28	1622-28D	1	1
314	427_S245_R1_001	427_29C-213A-5	427	1613-29×1622-29C	1613-29	1622-29C	1	1
315	428_S256_R1_001	428_29C-213A-6	428	1613-29×1622-29C	1613-29	1622-29C	1	1
316	432_S268_R1_001	432_3J-16A-3	432	1613-3×1622-3J	1613-3	1622-3J	1	1
317	435_S280_R1_001	435_30A-218A-5	435	1613-30×1622-30A	1613-30	1622-30A	1	1
318	436_S291_R1_001	436_30B-219A-4	436	1613-30×1622-30B	1613-30	1622-30B	1	1
319	437_S303_R1_001	437_30B-219A-7	437	1613-30×1622-30B	1613-30	1622-30B	1	1
320	438_S315_R1_001	438_30L-228A-4	438	1613-30×1622-30L	1613-30	1622-30L	1	1
321	439_S234_R1_001	439_30L-228A-3	439	1613-30×1622-30L	1613-30	1622-30L	1	1
322	440_S246_R1_001	440_31A-229A-3	440	1613-31×1622-31A	1613-31	1622-31A	1	1
323	441_S257_R1_001	441_31F-232A-5	441	1613-31×1622-31F	1613-31	1622-31F	1	1
324	442_S269_R1_001	442_31F-232A-6	442	1613-31×1622-31F	1613-31	1622-31F	1	1
325	446_S281_R1_001	446_5I-34A-4	446	1613-5×1622-5I	1613-5	1622-5I	1	1
326	447_S292_R1_001	447_5B-29A-3	447	1613-5×1622-5B	1613-5	1622-5B	1	1
327	449_S304_R1_001	449_5E-31A-5	449	1613-5×1622-5E	1613-5	1622-5E	1	1
328	450_S316_R1_001	450_5E-31A-6	450	1613-5×1622-5E	1613-5	1622-5E	1	1
329	451_S235_R1_001	451_5E-31A-7	451	1613-5×1622-5E	1613-5	1622-5E	1	1
330	452_S247_R1_001	452_5I-34A-3	452	1613-5×1622-5I	1613-5	1622-5I	1	1
331	453_S258_R1_001	453_6B-39A-6	453	1613-6×1622-6B	1613-6	1622-6B	1	1
332	454_S270_R1_001	454_6B-39A-4	454	1613-6×1622-6B	1613-6	1622-6B	1	1
333	456_S282_R1_001	456_6H-44A-4	456	1613-6×1622-6H	1613-6	1622-6H	1	1
334	457_S293_R1_001	457_7A-48A-3	457	1613-7×1622-7A	1613-7	1622-7A	1	1
335	459_S305_R1_001	459_9G-70A-4	459	1613-9×1622-9G	1613-9	1622-9G	1	1
336	461_S317_R1_001	461_10-76B-7	461	1622-10C×1613-10	1613-10	1622-10C	3	1
338	464_S329_R1_001	464_10-77B-5	464	1622-10D×1613-10	1613-10	1622-10D	1	1
339	465_S341_R1_001	465_10-77B-6	465	1622-10D×1613-10	1613-10	1622-10D	1	1
340	466_S353_R1_001	466_10-77B-4	466	1622-10D×1613-10	1613-10	1622-10D	1	1
341	467_S365_R1_001	467_10-79B-4	467	1622-10F×1613-10	1613-10	1622-10F	2	2
342	469_S377_R1_001	469_10-81B-7	469	1622-10K×1613-10	1613-10	1622-10K	1	1

343	470_S388_R1_001	470_10-81B-5	470	1622-10K×1613-10	1613-10	1622-10K	3	1
344	475_S400_R1_001	475_11-86B-7	475	1622-11F×1613-11	1613-11	1622-11F	1	1
345	476_S318_R1_001	476_11-86B-5	476	1622-11F×1613-11	1613-11	1622-11F	1	1
346	477_S330_R1_001	477_11-86B-4	477	1622-11F×1613-11	1613-11	1622-11F	1	1
347	478_S342_R1_001	478_13-102B-4	478	1622-13I×1613-13	1613-13	1622-13I	1	1
348	479_S354_R1_001	479_13-99B-4	479	1622-13F×1613-13	1613-13	1622-13F	1	1
349	480_S366_R1_001	480_13-99B-3	480	1622-13F×1613-13	1613-13	1622-13F	1	1
350	481_S378_R1_001	481_13-100B-3	481	1622-13G×1613-13	1613-13	1622-13G	1	1
351	482_S389_R1_001	482_13-101B-5	482	1622-13H×1613-13	1613-13	1622-13H	1	1
352	483_S401_R1_001	483_14-103B-4	483	1622-14A×1613-14	1613-14	1622-14A	1	1
353	485_S319_R1_001	485_14-103B-7	485	1622-14A×1613-14	1613-14	1622-14A	1	1
354	486_S331_R1_001	486_14-103B-5	486	1622-14A×1613-14	1613-14	1622-14A	1	1
355	487_S343_R1_001	487_15-108B-6	487	1622-15A×1613-15	1613-15	1622-15A	2	2
356	488_S355_R1_001	488_15-108B-7	488	1622-15A×1613-15	1613-15	1622-15A	2	2
357	489_S367_R1_001	489_15-108B-4	489	1622-15A×1613-15	1613-15	1622-15A	2	2
358	490_S379_R1_001	490_15-108B-3	490	1622-15A×1613-15	1613-15	1622-15A	2	2
359	492_S390_R1_001	492_15-108B-5	492	1622-15A×1613-15	1613-15	1622-15A	2	2
360	493_S402_R1_001	493_15-112B-5	493	1622-15F×1613-15	1613-15	1622-15F	2	2
361	494_S320_R1_001	494_15-112B-4	494	1622-15F×1613-15	1613-15	1622-15F	2	2
362	495_S332_R1_001	495_15-113B-3	495	1622-15G×1613-15	1613-15	1622-15G	2	2
363	496_S344_R1_001	496_15-113B-5	496	1622-15G×1613-15	1613-15	1622-15G	2	2
364	498_S356_R1_001	498_16-124B-4	498	1622-16I×1613-16	1613-16	1622-16I	2	2
365	499_S46_R1_001	499_16-119B-7	499	1622-16C×1613-16	1613-16	1622-16C	3	3
366	502_S380_R1_001	502_17-133B-3	502	1622-17I×1613-17	1613-17	1622-17I	1	1
367	503_S391_R1_001	503_17-133B-5	503	1622-17I×1613-17	1613-17	1622-17I	1	1
368	504_S403_R1_001	504_18-139B-5	504	1622-18C×1613-18	1613-18	1622-18C	3	3
369	505_S321_R1_001	505_18-139B-6	505	1622-18C×1613-18	1613-18	1622-18C	3	3
370	506_S333_R1_001	506_18-139B-7	506	1622-18C×1613-18	1613-18	1622-18C	3	3
371	507_S345_R1_001	507_18-139B-4	507	1622-18C×1613-18	1613-18	1622-18C	3	3
372	508_S357_R1_001	508_18-139B-3	508	1622-18C×1613-18	1613-18	1622-18C	3	3
373	509_S369_R1_001	509_18-140B-3	509	1622-18D×1613-18	1613-18	1622-18D	3	3
374	510_S381_R1_001	510_18-147B-4	510	1622-18L×1613-18	1613-18	1622-18L	3	3
375	511_S392_R1_001	511_18-147B-3	511	1622-18L×1613-18	1613-18	1622-18L	3	3
376	512_S404_R1_001	512_19-148B-4	512	1622-19B×1613-19	1613-19	1622-19B	1	1
377	513_S322_R1_001	513_19-153B-3	513	1622-19J×1613-19	1613-19	1622-19J	2	2
378	514_S334_R1_001	514_15-153B-4	514	1622-19J×1613-19	1613-19	1622-19J	1	1
379	515_S346_R1_001	515_20-160B-4	515	1622-20G×1613-20	1613-20	1622-20G	1	1
380	518_S358_R1_001	518_20-161B-3	518	1622-20H×1613-20	1613-20	1622-20H	1	1
381	519_S370_R1_001	519_20-164B-5	519	1622-20K×1613-20	1613-20	1622-20K	1	1
382	521_S382_R1_001	521_21-169B-3	521	1622-21G×1613-21	1613-21	1622-21G	1	1

383	522_S393_R1_001	522_21-169B-4	522	1622-21G×1613-21	1613-21	1622-21G	3	3
384	524_S405_R1_001	524_21-169B-7	524	1622-21G×1613-21	1613-21	1622-21G	3	3
385	525_S323_R1_001	525_21-171B-4	525	1622-21K×1613-21	1613-21	1622-21K	3	3
386	527_S335_R1_001	527_22-179B-3	527	1622-22I×1613-22	1613-22	1622-22I	1	1
387	528_S347_R1_001	528_22-176B-3	528	1622-22F×1613-22	1613-22	1622-22F	1	1
388	529_S359_R1_001	529_22-176B-4	529	1622-22F×1613-22	1613-22	1622-22F	1	1
389	530_S371_R1_001	530_22-176B-5	530	1622-22F×1613-22	1613-22	1622-22F	1	1
390	532_S383_R1_001	532_22-178B-4	532	1622-22H×1613-22	1613-22	1622-22H	2	2
391	533_S394_R1_001	533_22-178B-3	533	1622-22H×1613-22	1613-22	1622-22H	2	2
392	535_S406_R1_001	535_22-178B-6	535	1622-22H×1613-22	1613-22	1622-22H	2	2
393	536_S324_R1_001	536_23-182B-4	536	1622-23A×1613-23	1613-23	1622-23A	1	1
394	537_S336_R1_001	537_23-182B-6	537	1622-23A×1613-23	1613-23	1622-23A	1	1
395	540_S348_R1_001	540_23-183B-3	540	1622-23C×1613-23	1613-23	1622-23C	1	1
396	542_S360_R1_001	542_23-184B-4	542	1622-23D×1613-23	1613-23	1622-23D	1	1
397	543_S372_R1_001	543_23-184B-3	543	1622-23D×1613-23	1613-23	1622-23D	3	1
398	544_S384_R1_001	544_23-184B-7	544	1622-23D×1613-23	1613-23	1622-23D	1	1
399	548_S395_R1_001	548_26-194B-8	548	1622-26C×1613-26	1613-26	1622-26C	1	1
400	549_S407_R1_001	549_26-195B-4	549	1622-26D×1613-26	1613-26	1622-26D	2	2
401	550_S325_R1_001	550_26-196B-4	550	1622-26E×1613-26	1613-26	1622-26E	1	1
402	551_S337_R1_001	551_26-196B-5	551	1622-26E×1613-26	1613-26	1622-26E	3	3
403	552_S349_R1_001	552_26-196B-6-A	552	1622-26E×1613-26	1613-26	1622-26E	1	1
404	553_S361_R1_001	553_26-196B-6-B	553	1622-26E×1613-26	1613-26	1622-26E	2	2
405	555_S373_R1_001	555_26-201B-4	555	1622-26J×1613-26	1613-26	1622-26J	1	1
407	557_S396_R1_001	557_26-201B-3	557	1622-26J×1613-26	1613-26	1622-26J	2	2
408	558_S408_R1_001	558_28-206B-5	558	1622-28D×1613-28	1613-28	1622-28D	1	1
409	559_S326_R1_001	559_28-206B-7	559	1622-28D×1613-28	1613-28	1622-28D	1	1
410	561_S338_R1_001	561_28-211B-3	561	1622-28K×1613-28	1613-28	1622-28K	1	1
411	564_S350_R1_001	564_2-5B-5-B	564	1622-2E×1613-2	1613-2	1622-2E	1	1
412	565_S362_R1_001	565_2-5B-5-A	565	1622-2E×1613-2	1613-2	1622-2E	2	2
413	570_S374_R1_001	570_30-218B-4	570	1622-30A×1613-30	1613-30	1622-30A	1	1
414	572_S385_R1_001	572_20-223B-4	572	1622-30G×1613-30	1613-30	1622-30G	1	1
415	575_S397_R1_001	575_30-227B-5	575	1622-30K×1613-30	1613-30	1622-30K	1	1
416	576_S409_R1_001	576_30-227B-6	576	1622-30K×1613-30	1613-30	1622-30K	2	2
417	577_S327_R1_001	577_31-231B-3	577	1622-31D×1613-31	1613-31	1622-31D	1	1
418	581_S339_R1_001	581_3-10B-4	581	1622-3B×1613-3	1613-3	1622-3B	1	1
419	582_S351_R1_001	582_3-10B-3	582	1622-3B×1613-3	1613-3	1622-3B	1	1
420	584_S363_R1_001	584_3-11B-5	584	1622-3C×1613-3	1613-3	1622-3C	1	1
421	585_S375_R1_001	585_3-11B-7	585	1622-3C×1613-3	1613-3	1622-3C	1	1
422	586_S386_R1_001	586_3-13B-3	586	1622-3F×1613-3	1613-3	1622-3F	2	2
423	587_S398_R1_001	587_3-16B-5	587	1622-3J×1613-3	1613-3	1622-3J	1	1

424	588_S410_R1_001	588_3-16B-4	588	1622-3J×1613-3	1613-3	1622-3J	1	1
425	593_S328_R1_001	593_5-31B-7	593	1622-5E×1613-5	1613-5	1622-5E	1	1
426	594_S340_R1_001	594_5-31B-4	594	1622-5E×1613-5	1613-5	1622-5E	1	1
427	596_S352_R1_001	596_6-38B-7	596	1622-6A×1613-6	1613-6	1622-6A	1	1
428	598_S364_R1_001	598_6-41B-6	598	1622-6D×1613-6	1613-6	1622-6D	1	1
429	599_S376_R1_001	599_6-43B-3	599	1622-6G×1613-6	1613-6	1622-6G	2	2
430	600_S387_R1_001	600_7-48B-4	600	1622-7A×1613-7	1613-7	1622-7A	3	3
431	601_S399_R1_001	601_7-48B-3	601	1622-7A×1613-7	1613-7	1622-7A	1	1
432	605_S457_R1_001	605_9-69B-3	605	1622-9F×1613-9	1613-9	1622-9F	1	1
433	607_S469_R1_001	607_9-70B-4	607	1622-9G×1613-9	1613-9	1622-9G	1	1
434	608_S481_R1_001	608_23F-186A-2	608	1613-23×1622-23F	1613-23	1622-23F	1	1
435	609_S492_R1_001	609_29B-212A-2	609	1613-29×1622-29B	1613-29	1622-29B	1	1
436	603_S411_R1_001	603_7-49B-3	603	1622-7B×1613-7	1613-7	1622-7B	1	1

Table S3. Markers associated with yield biomass under drought stress. Phenotypic data were analyzed using three approaches: the single-stage model (ST1), the stage-wise approach averaged by month or year (ST2), and a model combining the first and last harvests of each year as fixed factors (DS). −log(p) correspond to −log10(p-value). PVE indicates the phenotypic variance explained.

Marker	Yield ST1	−log(p)	PVE	Yield ST2	−log(p)	PVE	DS	−log(p)	PVE
chr1.1_18947323	may_23	6.59	0.016	may;aug;hrv	6.77	−	2023	8.5	1.65E-04
chr1.1_31901620	sep_21	10.53	0.006	−	−	2.82E-03	2021	7.63	2.48E-04
chr1.1_45571686	sep_21	11.27	0.007	−	−	−	2021	6.7	3.34E-04
chr1.1_51052966	jun_20	7.68	0.006	−	−	−	−	−	−
chr1.1_63345275	sep_20	6.03	−	−	−	−	−	−	−
chr1.1_66113428	−	−	−	−	−	−	2021	6.15	2.52E-05
chr1.1_71650268	jul_23	6.92	0.004	−	−	−	−	−	−
chr2.1_1594541	−	−	−	may;aug;hrv	6.65	−	2023	7.77	5.20E-04
chr2.1_21245578	sep_21;jun_23	10.45	0.000	−	−	−	2021	7.77	1.74E-04
chr2.1_35177248	may_21	6.96	0.003	−	−	−	−	−	−
chr2.1_36991316	−	−	−	−	−	−	2022	5.99	1.45E-04
chr2.1_40029762	sep_21	5.86	0.020	sep	5.99	2.85E-03	−	−	−
chr2.1_41048095	aug_22	7.95	0.001	2022	6.91	7.37E-04	−	−	−
chr2.1_41372418	may_23	8.16	0.010	−	−	−	2023	6.09	−
chr2.1_41805885	jul_22;may_23	7.33	0.000	may;aug;hrv;2021;2022	6.65	−	2023	8.34	4.41E-04
chr2.1_42079284	jun_22;jun_22	7.86	0.045	−	−	−	2022	7.08	2.98E-06
chr2.1_42741704	−	−	−	−	−	−	2022	6.09	3.60E-04
chr2.1_43335071	jun_22	6.92	0.005	−	−	−	2022	6.95	2.89E-05
chr2.1_43634781	jun_22	7.19	0.002	−	−	−	−	−	−
chr2.1_45512985	jun_20	6.53	0.002	−	−	−	−	−	−
chr2.1_46357760	may_21	7.34	0.010	−	−	−	2021	6.28	−
chr2.1_46957218	may_23	8.08	0.041	−	−	−	2023	6.16	3.86E-04
chr2.1_5055117	may_21	8.73	0.046	may;2021	6.89	2.97E-02	2021	7.15	1.93E-03
chr2.1_56411272	jun_22	6.42	0.009	−	−	−	2022	6.4	8.46E-05
chr2.1_73298415	−	−	−	−	−	−	2021	6.12	−
chr3.1_1205458	sep_21;jun_23	10.85	0.000	sep	6.55	5.21E-03	2021	7.95	1.19E-05

chr3.1_14028345	may_23	6.76	–	–	–	–	–	–	–
chr3.1_24032838	may_23	7.41	0.058	–	–	–	–	–	–
chr3.1_24646587	may_23	7.52	0.010	–	–	–	2023	7.06	5.79E-06
chr3.1_24646759	may_23	6.75	0.020	–	–	–	2023	6.43	1.70E-04
chr3.1_28361901	sep_21;may_23	6.88	0.000	–	–	–	–	–	–
chr3.1_28470616	may_23	6.88	–	may;aug;hrv;2023	7.52	–	2023	8.3	3.47E-04
chr3.1_32059694	jul_22;jul_23	8.35	0.000	jul;aug;hrv;2021;2023	7.78	1.40E-14	2023	8.68	1.30E-04
chr3.1_33849324	sep_21	10.43	0.002	–	–	–	2021	7.79	4.25E-04
chr3.1_34280357	jun_22	6.17	–	–	–	–	2022	6.79	–
chr3.1_36512593	sep_21	11.1	0.000	–	–	–	2021	7.33	9.31E-04
chr3.1_46569537	jul_22	6.4	0.006	–	–	–	–	–	–
chr3.1_46589204	sep_21	6.16	0.002	–	–	–	–	–	–
chr3.1_55725039	may_23	8.83	0.005	–	–	–	2023	6.65	7.38E-04
chr3.1_55848695	may_23	6.98	0.002	–	–	–	2023	6.36	1.25E-03
chr3.1_56593873	sep_21;sep_21	8.47	0.022	–	–	–	–	–	–
chr3.1_56619522	may_23	6.69	0.020	–	–	–	–	–	–
chr3.1_58589185	sep_21	8.81	0.008	–	–	–	–	–	–
chr3.1_59145508	jun_20;sep_21	10.68	0.004	–	–	–	–	–	–
chr3.1_60492712	sep_21	7.29	0.003	–	–	–	–	–	–
chr3.1_6556553	sep_21	10.66	0.006	–	–	–	2021	7.83	9.65E-05
chr3.1_6563931	sep_21;jun_23	11.17	0.000	–	–	–	2021	8.44	8.01E-04
chr3.1_6563992	sep_21;jun_23	11.17	0.000	–	–	5.30E-03	2021	8.55	1.16E-04
chr3.1_80588990	jul_23	6.92	0.027	–	–	–	–	–	–
chr3.1_82158274	sep_21;sep_21	7.33	0.027	–	–	–	–	–	–
chr3.1_85101832	jun_21	6.37	0.006	–	–	–	–	–	–
chr4.1_10963532	jun_20	6	0.001	–	–	–	–	–	–
chr4.1_12219901	sep_21;jun_23	11.25	0.000	sep	6.7	3.76E-03	2021	8.56	8.55E-04
chr4.1_20948923	may_23	6.83	0.036	may;aug;hrv;2021;2022;2023	7.49	–	2023	8.64	4.65E-04
chr4.1_22418851	may_23	6.91	–	may;aug;hrv;2022;2023	7.51	–	2023	8.78	1.60E-04

chr4.1_26258871	–	–	–	–	–	–	2022	6.54	–
chr4.1_27636436	sep_21	6.32	–	–	–	–	–	–	–
chr4.1_2765855	–	–	–	–	–	–	2021	7.01	1.09E-03
chr4.1_36675681	jun_21	6.04	0.000	–	–	3.59E-02	–	–	–
chr4.1_36780567	may_23	8.69	0.016	–	–	–	2023	6.37	–
chr4.1_36780583	may_23	8.77	0.015	–	–	–	2023	6.38	–
chr4.1_41033612	may_23	6.63	–	may;aug;hrv;2022;2023	7.51	–	2023	8.73	3.51E-04
chr4.1_4716608	sep_21	6.03	0.035	–	–	–	–	–	–
chr4.1_47488164	aug_22;sep_22	6.07	0.000	–	–	–	–	–	5.95E-04
chr4.1_48930696	sep_21;jun_23	11.17	0.000	–	–	–	2021	8.49	3.25E-05
chr4.1_50772550	may_23	8.6	0.012	–	–	–	2023	6.27	–
chr4.1_60953306	–	–	0.030	–	–	–	2023	6.42	1.35E-04
chr4.1_79960120	may_23	9.7	0.048	–	–	–	2023	7.89	7.60E-04
chr4.1_88183831	–	–	–	–	–	–	2022	6.05	5.92E-06
chr5.1_1429434	–	–	–	–	–	–	2023	6.34	1.06E-03
chr5.1_21176923	sep_21	6.3	0.021	sep	6.86	1.94E-02	2020;2021	6.21	2.34E-04
chr5.1_2809364	sep_21	6.95	0.009	–	–	–	–	–	–
chr5.1_29813485	may_23	8.74	0.021	–	–	–	2023	6.67	1.22E-03
chr5.1_42131042	sep_21	10.02	0.013	sep	5.97	8.72E-05	–	–	–
chr5.1_46799633	may_23	8.68	0.017	–	–	–	2023	6.52	–
chr5.1_51671055	sep_21;jun_23	11.44	0.060	–	–	–	2021	7.99	8.09E-04
chr5.1_64373063	may_23	9.54	0.006	–	–	–	2023	6.95	9.58E-05
chr5.1_68906404	may_23	8.17	0.006	–	–	–	2023	6.05	6.29E-05
chr5.1_70363180	sep_21	10.49	0.027	–	–	–	2021	7.43	6.82E-04
chr5.1_71961988	jun_23	6.02	–	–	–	–	–	–	–
chr6.1_1192177	may_23	6.02	–	–	–	–	–	–	–
chr6.1_12947000	–	–	–	jun	6.04	2.59E-03	–	–	–
chr6.1_12947881	sep_22;jun_23	6.55	0.022	jun	6.05	5.10E-03	–	–	–
chr6.1_22955180	sep_21	6.1	0.003	–	–	6.93E-03	–	–	–

chr6.1_27950240	aug_20;jun_22;jul_22;aug_22;may_23	9.76	–	may;aug;sep;hrv;2020;2022	8.5	–	2022	7.27	–
chr6.1_39491281	–	–	–	–	–	–	2021	6.32	–
chr6.1_43709038	sep_20	6.02	0.001	–	–	–	–	–	–
chr6.1_49779667	sep_21	8.2	0.008	sep	7.01	9.76E-03	2021	6.49	–
chr6.1_54561217	may_21	6.93	–	–	–	–	–	–	–
chr6.1_67638734	sep_21	6.84	–	–	–	–	–	–	–
chr6.1_71935085	may_23	8.73	0.017	–	–	–	2023	6.94	–
chr6.1_71935145	may_23	8.73	0.028	–	–	–	2023	6.7	1.63E-03
chr6.1_71963797	may_23	9.62	0.023	–	–	–	2023	7.77	6.30E-04
chr6.1_72523411	jul_23	7.42	–	–	–	–	–	–	–
chr6.1_72523573	jul_23;aug_23	7.38	0.000	–	–	–	–	–	–
chr6.1_78855038	jul_21	6.3	–	–	–	–	–	–	–
chr7.1_11234756	may_23	7.05	–	may;aug;hrv;2022;2023	8.06	–	2022;2023	8.86	7.93E-04
chr7.1_12367626	may_23	9.21	0.020	–	–	–	2023	6.54	2.93E-04
chr7.1_12556475	sep_21	6.02	0.005	–	–	–	–	–	–
chr7.1_13004225	aug_22	6.7	0.002	–	–	–	–	–	–
chr7.1_13900254	sep_21;sep_21	7.81	0.008	–	–	–	–	–	–
chr7.1_13903585	jun_20;sep_21	10.79	0.000	–	–	–	–	–	–
chr7.1_16017211	sep_21;jun_23	10.95	0.012	–	–	–	2021	8.11	9.24E-05
chr7.1_16042206	sep_21;jun_23	11.09	0.007	sep	6.12	–	2021	8.58	2.28E-04
chr7.1_16625178	sep_21;jun_23	10.28	0.000	–	–	–	2021	8.17	1.12E-04
chr7.1_19253703	jun_20;sep_21	10.66	0.013	–	–	–	–	–	–
chr7.1_19458707	sep_21	6.97	–	–	–	–	–	–	–
chr7.1_21096065	may_23	8.13	0.009	–	–	–	2023	6.4	3.13E-04
chr7.1_21709512	sep_21	9.59	0.000	–	–	–	2021	7.13	2.29E-04
chr7.1_22042287	may_23	8.69	0.019	–	–	–	2023	6.55	–
chr7.1_22743142	may_20	6.21	0.005	–	–	–	2020	6.85	6.37E-04
chr7.1_24014763	jun_20;sep_21	9.99	0.044	–	–	–	–	–	–
chr7.1_27529705	jun_20;sep_21	10.73	0.022	–	–	–	–	–	–

chr7.1_27530944	jun_20;sep_21	10.7	0.020	–	–	–	–	–	–
chr7.1_28073304	sep_21	9.13	0.006	jun;sep	8.19	4.84E-04	2021	6.25	–
chr7.1_3099206	aug_22	6.07	–	–	–	–	–	–	–
chr7.1_32499357	may_23	6.92	–	may;aug;hrv;2022;2023	7.41	–	2023	8.96	2.49E-04
chr7.1_33215726	may_23	8.63	0.010	–	–	–	2023	6.45	–
chr7.1_34608004	sep_21;jun_23	11.18	0.020	sep	6.04	–	2021	8.26	2.97E-04
chr7.1_35070612	–	–	–	–	–	–	2023	6.38	4.45E-04
chr7.1_39036220	may_23	8.82	0.033	–	–	–	2023	6.48	–
chr7.1_57264444	may_23	8.65	0.023	–	–	–	2023	6.44	–
chr7.1_66487639	jun_20;sep_20	10.92	0.000	sep;2020	8.27	–	2020	7.64	5.64E-03
chr7.1_67995967	may_23	6.88	0.081	–	–	–	–	–	–
chr7.1_9018477	sep_21;sep_21	7.14	0.000	–	–	–	–	–	–
chr7.1_9637543	may_21	7.42	0.043	–	–	–	–	–	–
chr8.1_28345237	may_23	8.93	0.031	–	–	–	2023	6.46	–
chr8.1_31427565	may_23	8.71	0.022	–	–	–	2023	6.53	–
chr8.1_39745739	sep_21	7.14	0.029	–	–	–	–	–	–
chr8.1_43217387	sep_21	9.51	0.022	–	–	–	–	–	–
chr8.1_52715690	sep_21	9.82	0.019	–	–	–	–	–	–
chr8.1_61331375	sep_21;sep_21	8.98	0.029	–	–	–	2021	6.55	4.03E-04
chr8.1_72832758	–	–	–	–	–	–	2021	7.05	1.40E-03
chr8.1_81622742	jul_22	6.13	0.071	–	–	–	–	–	–
chr8.1_84752525	may_23	9.37	0.003	–	–	–	2023	6.94	5.23E-04
chr8.1_84778085	may_23	8.95	0.011	–	–	–	2023	6.8	4.65E-04
chr8.1_9331478	sep_20	6.23	–	–	–	–	–	–	–

Table S4. Marker annotations and gene information. Genes located at the same positions as the markers were annotated. Gene annotations, including protein names and functions obtained from the UniProt database [2]. C corresponds to chromosome. PK, protein kinase; CW, cell wall development; TF, transcription factor; Ion, Ion transport and homeostasis.

Marker	C	Position	UniProt	Protein names	Gene	Class
chr1.1_31901620	1	31901620	UPI000CE223E1	Ubiquitin carboxyl-terminal hydrolase 12		
chr1.1_45571686	1	45571686	UPI000CE26C9A	Uncharacterized protein		
chr1.1_51052966	1	51052966	A0A445IIA7	beta-ketoacyl-[acyl-carrier-protein] synthase I		
chr1.1_66113428	1	66113428	A0A4D6N0L2	P-loop containing nucleoside triphosphate hydrolase		
chr1.1_71650268	1	71650268	UPI00098DB915	Putative cell division cycle ATPase		ATPase
chr2.1_5055117	2	5055117	A0A4Y1R404	Translocase of inner mitochondrial membrane 23		
chr2.1_21245578	2	21245578	A0A0L9UM16	Phytocyanin domain-containing protein		
chr2.1_36991316	2	36991316	A0A2P5C7B9	Serine-threonine kinase receptor-associated protein	*STRAP*	
chr2.1_40029762	2	40029762	A0A445AVH9	Reticulon-like protein		
chr2.1_41048095	2	41048095	A0A2P5AKY0	P-type ATPase		ATPase
chr2.1_42079284	2	42079284	A0A2I4G7M1	osteosarcoma-9 homolog isoform X2	OS-9	
chr2.1_46357760	2	46357760	A0A392MY71	Putative E3 ubiquitin-protein ligase RNF217-like		
chr2.1_46957218	2	46957218	A0A5B8TZ92	Sucrose synthase	*SuSin*	CW
chr3.1_24032838	3	24032838	UPI00092ED1AF	Choline transporter-like protein		
chr3.1_28470616	3	28470616	UPI0010A32E32	Uncharacterized protein		
chr3.1_33849324	3	33849324	A0A0R0IV15	Nucleic acid binding NABP domain-containing protein		
chr3.1_34280357	3	34280357	UPI000F7C96BE	Receptor-like protein EIX2		
chr3.1_46569537	3	46569537	A0A396ISR4	Uncharacterized protein		
chr3.1_55725039	3	55725039	UPI000D627F00	Proline-rich receptor-like protein kinase 13	*PERK13*	PK
chr3.1_55848695	3	55848695	UPI00092F5E8D	Protease Do-like 7		
chr3.1_56593873	3	56593873	G7KGV6	Mannose-1-phosphate guanylyltransferase	*ManB*	CW
chr3.1_56619522	3	56619522	G7J9Z6	Folylpolyglutamate synthase		
chr3.1_58589185	3	58589185	A0A5J5AUQ5	ATP-dependent RNA helicase		
chr3.1_58589185	3	58589185	I3SUM8	Transmembrane protein		
chr3.1_80588990	3	80588990	UPI0010A3306E	Uncharacterized protein		
chr3.1_85101832	3	85101832	A0A1S2Y942	Overexpressor of cationic peroxidase 3	*OPC3*	TF
chr4.1_2765855	4	2765855	A0A2C9UB23	COV1-like protein		
chr4.1_4716608	4	4716608	UPI00077E5CE8	KAT8 regulatory NSL complex subunit 3		
chr4.1_4716608	4	4716608	UPI00092EBF23	Uncharacterized protein		
chr4.1_10963532	4	10963532	A0A0S3QYN3	Pentacotripeptide-repeat region of PRORP domain	*PPR*	
chr4.1_12219901	4	12219901	UPI000DED13FC	YTH domain-containing protein ECT4		
chr4.1_20948923	4	20948923	A0A445I6C2	Vesicle-fusing ATPase		ATPase
chr4.1_27636436	4	27636436	A0A5A7V2I4	ABC transporter G family member 15-like		Ion
chr4.1_36675681	4	36675681	A0A0L9UNF0	Putative E3 ubiquitin-protein		
chr4.1_41033612	4	41033612	A0A0D2N8F9	C2H2-type domain-containing protein	*C2H2*	TF

chr4.1_47488164	4	47488164	B7FKW2	Transmembrane protein		Ion
chr4.1_48930696	4	48930696	UPI000A2B440C	RRP6-like 3		
chr4.1_50772550	4	50772550	UPI0007AF7205	Zinc finger CCCH domain-containing protein 13	*C3H*	TF
chr4.1_60953306	4	60953306	A0A445E1Y7	RING-type domain-containing protein		
chr4.1_88183831	4	88183831	UPI000CE1F86E	Putative cyclic nucleotide-gated ion channel 8		Ion
chr4.1_88183831	4	88183831	A0A4U5PLL7	DUF1995 domain-containing protein		
chr5.1_1429434	5	1429434	A0A445D778	NADH:flavin oxidoreductase	*OR*	
chr5.1_1429434	5	1429434	UPI000CE18E26	12-oxophytodienoate reductase 2	*OPDA*	
chr5.1_2809364	5	2809364	UPI000CED42D0	Cycloartenol synthase 2	*CAS2*	
chr5.1_29813485	5	29813485	UPI000B3F366F	Transcription factor TCP15	*TCP15*	TF
chr5.1_42131042	5	42131042	A0A1J7GNY4	Phospholipid-transporting ATPase		ATPase
chr5.1_46799633	5	46799633	A0A151TQE0	Transcription factor TGA7	*TGA7*	TF
chr5.1_51671055	5	51671055	UPI000848C0E7	Uncharacterized protein		
chr5.1_64373063	5	64373063	A0A0S3T1U6	60S acidic ribosomal protein P0		
chr5.1_68906404	5	68906404	UPI000D2F1616	Uncharacterized protein		
chr5.1_71961988	5	71961988	UPI000F7CBE24	DExH-box ATP-dependent RNA helicase DExH6		
chr6.1_1192177	6	1192177	UPI00046E0890	Mitogen-activated protein kinase kinase 3	MAPKK3	PK
chr6.1_27950240	6	27950240	A0A2P6SND3	Membrane protein YjcL		
chr6.1_43709038	6	43709038	UPI0010166E77	Nodulin/glutamine synthase-like protein	*NODGS*	
chr6.1_54561217	6	54561217	UPI000CED42D3	K(+) efflux antiporter 5	*KEA5*	
chr6.1_67638734	6	67638734	A0A5B7BD01	DHHA1 domain-containing protein		
chr6.1_71935085	6	71935085	UPI000F7C35A4	Cyclin-dependent kinase D-3-like	*CDK3*	PK
chr6.1_78855038	6	78855038	E1U3U6	Zinc finger protein ZF3	*C3H*	TF
chr7.1_3099206	7	3099206	UPI000809BEC7	Uncharacterized protein		
chr7.1_9637543	7	9637543	A0A5B6ZTS3	VPS37 C-terminal domain-containing protein		
chr7.1_12367626	7	12367626	UPI00053C353D	BI1-like protein		
chr7.1_13903585	7	13903585	A0A1S4DUQ7	Uncharacterized protein		
chr7.1_16017211	7	16017211	A0A2I4GD16	Uncharacterized protein		
chr7.1_16042206	7	16042206	UPI000B7B92CC	Pentatricopeptide repeat-containing protein	*PPR*	
chr7.1_19458707	7	19458707	A0A1R3GW37	Glutamate dehydrogenase	*GDH*	
chr7.1_21096065	7	21096065	UPI001004D2FE	Uncharacterized protein		
chr7.1_22042287	7	22042287	UPI000DED0270	Eukaryotic translation initiation factor 5		
chr7.1_22743142	7	22743142	A0A371I8J1	Serine/threonine protein kinase	*D6PKL2*	PK
chr7.1_24014763	7	24014763	A0A1J7IXN9	Rad21/Rec8-like protein N-terminal		
chr7.1_24014763	7	24014763	A0A5B6ZJC0	Prokaryotic-type class I peptide chain release factors		
chr7.1_27529705	7	27529705	UPI0007192AEA	BEACH domain-containing protein C2-like		
chr7.1_32499357	7	32499357	A0A1S3TA63	Ammonium transporter		Ion
chr7.1_33215726	7	33215726	A0A371GZH7	Calcium-dependent protein kinase 13	*CDPK13*	PK
chr7.1_34608004	7	34608004	A0A445EVW4	Pentatricopeptide repeat-containing protein	*PPR*	

chr7.1_34608004	7	34608004	B7FIB1	Transmembrane protein		Ion
chr7.1_39036220	7	39036220	UPI000809BA42	Auxin response factor 9	*ARF9*	TF
chr7.1_66487639	7	66487639	A0A0L9TSI7	GTP-binding protein		
chr7.1_67995967	7	67995967	A0A445EV80	Bms1-type G domain-containing protein		
chr8.1_28345237	8	28345237	A0A2K3NA96	Putative disease resistance protein		
chr8.1_31427565	8	31427565	UPI0005111147	Uncharacterized protein		
chr8.1_43217387	8	43217387	A0A2K1J132	Histone H3		
chr8.1_52715690	8	52715690	UPI000F7C66E1	Ubiquitin-conjugating enzyme E2 25		
chr8.1_61331375	8	61331375	UPI000B76DB48	Uncharacterized protein		
chr8.1_72832758	8	72832758	A0A0S3SZ85	Pectate lyase superfamily protein	*PEL*	
chr8.1_72832758	8	72832758	UPI00103640C0	Mitogen-activated protein kinase	*MAPK2*	PK
chr8.1_84752525	8	84752525	A0A1S3C891	Xyloglucan glycosyltransferase 6	*XGT6*	CW

Table S5. Candidate genes prioritized by markers associated with drought stress (DS) of alfalfa within a window of 2 Mb. Dist is the distance from the marker to the gene in kb, where d and u correspond to downstream or upstream position, respectively; r_{pb} is the point biserial correlation coefficient, indicating whether the gene has significantly increased or decreased expression levels in DS; Function include Dev, plant development; DS, drought stress; Cold, cold stress; TF, transcription factor, H, hub gene according to gene coexpression network [3].

Marker	Uniprot	Dist	r_{pb}	Protein name	Gene	Function	Refs
chr1.1_18947323	A0A2I4FVC9	74 d	-0.47	Armadillo repeat-containing protein 6	*ARMC6*	Dev	[4]
chr1.1_63345275	A0A0L9TYJ0	760 d	0.5	Hexosyltransferase		H	
chr1.1_66113428	A0A0L9VKW0	898 d	-0.58	Indole-3-acetic acid-induced protein	*ARG2*	DS	[5]
chr1.1_66113428	A0A498JB63	22 d	0.52	Exocyst subunit Exo70 family protein	*EXO70*	DS	[6]
chr1.1_71650268	UPI00103C9E63	842 u	0.45	Low-temperature-induced 65 kDa protein-	*LTI65*	Cold, DS	[7]
chr2.1_5055117	A0A394DGZ2	429 d	-0.55	Late embryogenesis abundant protein LEA-2	*LEA II*	Cold, DS	[8]
chr2.1_5055117	UPI000CE26090	101 u	0.55	Glycine-rich RNA-binding protein 2	*GRP2*	Cold, DS	[9]
chr2.1_56411272	A0A2I4ESV4	952 u	-0.57	Pathogenesis-related protein 1-like	*PR1*		
chr2.1_73298415	F6I738	962 d	0.53	MYB102 transcription factor	*MYB102*	TF, DS, H	[10]
chr3.1_6556553	A0A2Z6MHP5	94 d	-0.61	F-box domain-containing protein			
chr3.1_6563931	A0A2Z6MHP5	102 d	-0.61	F-box domain-containing protein			
chr3.1_6563992	A0A2Z6MHP5	102 d	-0.61	F-box domain-containing protein			
chr3.1_46569537	A0A0R0IW87	5 u	0.5	Maternal effect embryo arrest 59	*MEE59*	H	
chr3.1_46589204	A0A0R0IW87	15 d	0.5	Maternal effect embryo arrest 59	*MEE59*	H	
chr3.1_46569537	A0A2G9I2I1	21 d	0.56	Proliferating cell nuclear antigen	*PCNA*		
chr3.1_46589204	A0A2G9I2I1	41 d	0.56	Proliferating cell nuclear antigen	*PCNA*		
chr3.1_55725039	A0A565CMH8	236 u	0.63	Cold-regulated protein	*COR*	Cold, H	[11]
chr3.1_55848695	A0A565CMH8	113 u	0.63	Cold-regulated protein	*COR*	Cold, H	[11]
chr3.1_58589185	A0A453EFA1	404 d	0.48	Histone H4			
chr3.1_58589185	I1JID6	47 u	-0.61	Abscisic acid receptor PYL4	*PYL4*	DS	[12]
chr3.1_60492712	A0A151TZQ1	280 u	-0.44	DnaJ isogeny subfamily C member 7			
chr3.1_82158274	A0A1R3GUV3	489 u	0.45	EngB-type G domain-containing protein			
chr4.1_2765855	A0A5B7C5D2	397 u	0.46	Serine/threonine phosphatase			
chr4.1_4716608	UPI000F7CEAA5	192 d	0.56	Dynamin-related protein 5A-like isoform X1			
chr4.1_4716608	A0A5E4FW16	722 u	-0.59	Polygalacturonase			

chr4.1_20948923	A0A453PBR0	613 u	0.52	Uncharacterized protein			
chr4.1_22418851	A0A453MZP2	261 d	0.52	Histone H3.2		H	
chr4.1_22418851	V7CI09	913 u	-0.64	Alpha/beta hydrolase fold-3 domain			
chr4.1_27636436	A0A1U8BJB4	399 d	-0.46	Prohibitin	*PHB*	Dev	[13]
chr4.1_36780567	I3SL45	425 u	0.49	Transmembrane protein			
chr4.1_60953306	UPI000D2F378C	872 u	-0.5	Uncharacterized protein			
chr4.1_88183831	A0A4U5QI13	276 d	0.47	DNAJ heat shock N-terminal domain		H	
chr5.1_1429434	A0A2R6Q9J9	920 d	-0.58	GPCR-type G protein	*GPCR*		
chr5.1_1429434	A0A251QR34	251 d	-0.49	VOC domain-containing protein	*VOC*		
chr5.1_1429434	A0A2J6JWC6	383 d	-0.57	PABS domain-containing protein	*PABS*		
chr5.1_21176923	UPI000786B184	588 u	-0.48	7-deoxyloganetic acid glucosyltransferase	*7DLGT*	H	
chr5.1_71961988	UPI000C209F76	557 u	0.47	4-hydroxyphenylpyruvate dioxygenase	*HPPD*	DS	[14]
chr6.1_1192177	A0A2N9I7M1	578 d	-0.51	Glucose-6-phosphate 1-epimerase			
chr6.1_1192177	A0A345AM08	217 d	0.68	Galactinol-sucrose galactosyltransferase		H	
chr6.1_54561217	A0A540KI08	107 d	0.5	RNA helicase			
chr6.1_72523411	A0A2K3NNM3	71 d	-0.46	Uncharacterized protein			
chr6.1_72523573	A0A2K3NNM3	71 d	-0.46	Uncharacterized protein			
chr7.1_3099206	A0A4P1QUT6	55 u	0.55	PWWP domain-containing protein			
chr7.1_9018477	K7VJZ5	307 u	-0.5	Aquaporin	*TIP1:1*	DS	[15]
chr7.1_9637543	K7VJZ5	312 d	-0.5	Aquaporin	*TIP1:1*	DS	[15]
chr7.1_16625178	A0A445CM34	569 u	0.44	Pyrroline-5-carboxylate reductase	*P5CR*	DS	[16]
chr7.1_19253703	A0A4Y7L4V3	450 d	-0.46	Cyclin-like domain-containing protein			
chr7.1_19253703	A0A1J7HFW6	250 u	-0.56	Inosine-5'-monophosphate dehydrogenase	*IMPDH*		
chr7.1_19458707	A0A1J7HFW6	45 u	-0.56	Inosine-5'-monophosphate dehydrogenase	*IMPDH*		
chr7.1_19253703	UPI0010A44974	442 u	0.49	Thioredoxin-like 1-1, chloroplastic	*TRX*		
chr7.1_19458707	UPI0010A44974	237 u	0.49	Thioredoxin-like 1-1, chloroplastic	*TRX*		
chr7.1_21096065	UPI0011D282AD	64 u	0.44	PXR1			
chr7.1_21709512	UPI0011D282AD	549 d	0.44	PXR1			
chr7.1_28073304	A0A0B0PD94	588 u	-0.44	KH domain-containing protein			

chr7.1_32499357	I1JL81	289 d	0.44	Ferritin		H	
chr7.1_67995967	I3SD40	940 u	-0.47	Squamosa promoter binding protein SBP TF	*SBP*	TF, Dev	[17]
chr8.1_9331478	V7BS64	237 d	0.53	CASP-like protein			
chr8.1_39745739	A0A2H3XS40	714 d	-0.53	Uncharacterized protein			
chr8.1_52715690	A0A445DYH3	398 d	0.47	Phytocyanin domain-containing protein			
chr8.1_52715690	UPI00077E7A59	576 u	0.45	Translocon Sec61/SecY plug domain			

Table S6. Predicted abilities (PA) of genomic best linear unbiased prediction (GBLUP) model using different genomic relationship matrices (GRM). PA correspond to mean values of 10 iterations with 10-fold cross-validation and values in brackets correspond to the ranges. PA were calculated as Pearson's correlation between genomic estimated breeding values and phenotypes of test population for yield in 18 harvests or by yield averaged by month (ST2-M), year (ST2-Y), or overall yield (bold).

Harvest	2020	2021	2022	2023	ST2-M
**G1**					
May	0.15 (0.12-0.19)	0.16 (0.13-0.19)	–	-0.07 (-0.11-0.01)	0.18 (0.14-0.21)
Jun	0.09 (0.06-0.12)	0.12 (0.08-0.17)	0.11 (0.06-0.14)	0.06 (0.02-0.11)	0.18 (0.15-0.21)
Jul	0.12 (0.08-0.15)	0.05 (0-0.07)	0.1 (0.05-0.12)	0.12 (0.08-0.15)	0.19 (0.16-0.22)
Aug	0.13 (0.11-0.15)	0.07 (0.03-0.11)	0.05 (0.02-0.1)	0.15 (0.11-0.17)	0.18 (0.16-0.2)
Sep	0.12 (0.08-0.16)	0.12 (0.1-0.16)	0.07 (0.01-0.1)	–	0.19 (0.16-0.23)
**ST2-Y**	0.19 (0.16-0.23)	0.17 (0.15-0.19)	0.16 (0.12-0.19)	0.19 (0.16-0.22)	**0.2 (0.17-0.23)**
**G2**					
May	0.53 (0.51-0.54)	0.6 (0.59-0.62)	–	0.44 (0.42-0.46)	0.74 (0.73-0.75)
Jun	0.6 (0.59-0.62)	0.55 (0.54-0.58)	0.46 (0.45-0.47)	0.44 (0.42-0.45)	0.79 (0.78-0.8)
Jul	0.45 (0.42-0.47)	0.59 (0.58-0.61)	0.51 (0.5-0.52)	0.42 (0.4-0.44)	0.78 (0.77-0.79)
Aug	0.57 (0.56-0.58)	0.49 (0.47-0.51)	0.39 (0.38-0.41)	0.34 (0.3-0.36)	0.77 (0.76-0.78)
Sep	0.47 (0.44-0.49)	0.4 (0.39-0.41)	0.44 (0.43-0.45)	–	0.74 (0.73-0.76)
**ST2-Y**	0.76 (0.75-0.77)	0.75 (0.74-0.76)	0.73 (0.72-0.74)	0.78 (0.77-0.79)	**0.83 (0.82-0.84)**
**G3**					
May	0.59 (0.58-0.6)	0.65 (0.65-0.67)	–	0.51 (0.49-0.54)	0.79 (0.78-0.8)
Jun	0.67 (0.65-0.68)	0.62 (0.61-0.64)	0.52 (0.51-0.54)	0.5 (0.48-0.51)	0.86 (0.85-0.87)
Jul	0.53 (0.51-0.54)	0.66 (0.64-0.67)	0.57 (0.56-0.58)	0.48 (0.46-0.49)	0.84 (0.83-0.84)
Aug	0.64 (0.62-0.65)	0.57 (0.55-0.58)	0.48 (0.47-0.49)	0.38 (0.34-0.4)	0.82 (0.81-0.83)
Sep	0.51 (0.48-0.52)	0.48 (0.46-0.49)	0.51 (0.5-0.51)	–	0.79 (0.78-0.8)
**ST2-Y**	0.82 (0.81-0.83)	0.8 (0.79-0.81)	0.77 (0.76-0.78)	0.84 (0.83-0.85)	**0.9 (0.89-0.9)**
**G4**					
May	0.58 (0.57-0.59)	0.66 (0.64-0.67)	–	0.51 (0.49-0.53)	0.78 (0.77-0.79)
Jun	0.66 (0.65-0.67)	0.62 (0.61-0.64)	0.53 (0.51-0.54)	0.48 (0.47-0.49)	0.86 (0.85-0.86)
Jul	0.52 (0.51-0.54)	0.66 (0.64-0.67)	0.57 (0.55-0.58)	0.47 (0.46-0.49)	0.84 (0.83-0.84)
Aug	0.63 (0.61-0.64)	0.57 (0.56-0.58)	0.48 (0.47-0.49)	0.37 (0.33-0.4)	0.82 (0.81-0.82)
Sep	0.5 (0.48-0.52)	0.48 (0.46-0.49)	0.5 (0.49-0.51)	–	0.79 (0.78-0.8)
**ST2-Y**	0.82 (0.81-0.83)	0.8 (0.79-0.81)	0.77 (0.76-0.78)	0.83 (0.83-0.85)	**0.89 (0.89-0.9)**

References

1. Rodríguez-Álvarez MX, Boer MP, van Eeuwijk FA, Eilers PHC. Spatial Models for Field Trials. 2016; 1–39. Available: http://arxiv.org/abs/1607.08255
2. Bateman A. UniProt: a worldwide hub of protein knowledge. Nucleic Acids Res. 2019;47: D506–D515. doi:10.1093/nar/gky1049
3. Almeida-Silva F, Venancio TM. *cageminer*: an R/Bioconductor package to prioritize candidate genes by integrating genome-wide association studies and gene coexpression

networks. Marshall-Colon A, editor. In Silico Plants. 2022;4. doi:10.1093/insilicoplants/diac018
4. Coates JC, Laplaze L, Haseloff J. Armadillo-related proteins promote lateral root development in Arabidopsis. Proc Natl Acad Sci U S A. 2006;103. doi:10.1073/pnas.0507575103
5. Zhang Y, Li Y, Hassan MJ, Li Z, Peng Y. Indole-3-acetic acid improves drought tolerance of white clover via activating auxin, abscisic acid and jasmonic acid related genes and inhibiting senescence genes. BMC Plant Biol. 2020;20. doi:10.1186/s12870-020-02354-y
6. Ogura T, Goeschl C, Filiault D, Mirea M, Slovak R, Wolhrab B, et al. Root System Depth in Arabidopsis Is Shaped by EXOCYST70A3 via the Dynamic Modulation of Auxin Transport. Cell. 2019;178. doi:10.1016/j.cell.2019.06.021
7. Nordin K, Vahala T, Palva ET. Differential expression of two related, low-temperature-induced genes in Arabidopsis thaliana (L.) Heynh. Plant Mol Biol. 1993;21. doi:10.1007/BF00014547
8. Magwanga RO, Lu P, Kirungu JN, Lu H, Wang X, Cai X, et al. Characterization of the late embryogenesis abundant (LEA) proteins family and their role in drought stress tolerance in upland cotton. BMC Genet. 2018;19. doi:10.1186/s12863-017-0596-1
9. Kim JY, Park SJ, Jang B, Jung CH, Ahn SJ, Goh CH, et al. Functional characterization of a glycine-rich RNA-binding protein 2 in Arabidopsis thaliana under abiotic stress conditions. Plant Journal. 2007;50. doi:10.1111/j.1365-313X.2007.03057.x
10. Piao W, Sakuraba Y, Paek NC. Transgenic expression of rice MYB102 (OsMYB102) delays leaf senescence and decreases abiotic stress tolerance in Arabidopsis. BMB Rep. 2019;52. doi:10.5483/BMBRep.2019.52.11.071
11. Thomashow MF. Plant cold acclimation: Freezing tolerance genes and regulatory mechanisms. Annu Rev Plant Biol. 1999;50. doi:10.1146/annurev.arplant.50.1.571
12. Lackman P, González-Guzmán M, Tilleman S, Carqueijeiro I, Pérez AC, Moses T, et al. Jasmonate signaling involves the abscisic acid receptor PYL4 to regulate metabolic reprogramming in *Arabidopsis* and tobacco. Proceedings of the National Academy of Sciences. 2011;108: 5891–5896. doi:10.1073/pnas.1103010108
13. Huang R, Yang C, Zhang S. The Arabidopsis PHB3 is a pleiotropic regulator for plant development. Plant Signaling and Behavior. 2019. doi:10.1080/15592324.2019.1656036
14. Kim SE, Bian X, Lee CJ, Park SU, Lim YH, Kim BH, et al. Overexpression of 4-hydroxyphenylpyruvate dioxygenase (IbHPPD) increases abiotic stress tolerance in transgenic sweetpotato plants. Plant Physiology and Biochemistry. 2021;167. doi:10.1016/j.plaphy.2021.08.025
15. Shivaraj SM, Sharma Y, Chaudhary J, Rajora N, Sharma S, Thakral V, et al. Dynamic role of aquaporin transport system under drought stress in plants. Environmental and Experimental Botany. 2021. doi:10.1016/j.envexpbot.2020.104367
16. Chen C, Cui X, Zhang P, Wang Z, Zhang J. Expression of the pyrroline-5-carboxylate reductase (P5CR) gene from the wild grapevine Vitis yeshanensis promotes drought resistance in transgenic Arabidopsis. Plant Physiology and Biochemistry. 2021;168. doi:10.1016/j.plaphy.2021.10.004
17. Ma L, Liu X, Liu W, Wen H, Zhang Y, Pang Y, et al. Characterization of Squamosa-Promoter Binding Protein-Box Family Genes Reveals the Critical Role of MsSPL20 in Alfalfa Flowering Time Regulation. Front Plant Sci. 2022;12. doi:10.3389/fpls.2021.775690
